# Integrated proteogenomic characterization reveals an imbalanced hepatocellular carcinoma microenvironment after incomplete radiofrequency ablation

**DOI:** 10.1186/s13046-023-02716-y

**Published:** 2023-05-25

**Authors:** Zheng-Rong Shi, Yu-Xin Duan, Fang Cui, Zhong-Jun Wu, Mao-Ping Li, Pei-Pei Song, Qi-Ling Peng, Wen-Tao Ye, Kun-Li Yin, Mei-Qing Kang, Yan-Xi Yu, Jian Yang, Wei Tang, Rui Liao

**Affiliations:** 1 Department of Hepatobiliary Surgery, The First Affiliated Hospital of Chongqing Medical University, No. Youyi Rd, Chongqing, 400016 China; 2 Department of Laboratory Medicine, The First Affiliated Hospital of Chongqing Medical University, Chongqing, China; 3 Department of Ultrasound, The First Affiliated Hospital of Chongqing Medical University, Chongqing, China; 4 National Center for Global Health and Medicine, Tokyo, Japan; 5 Faculty of Basic Medical Sciences, Chongqing Medical University, Chongqing, 400016 China

**Keywords:** Liver cancer, Thermal ablation, Omics analysis, Immune response, Proteinase 3

## Abstract

**Background:**

Efforts to precisely assess tumor-specific T-cell immune responses still face major challenges, and the potential molecular mechanisms mediating hepatocellular carcinoma (HCC) microenvironment imbalance after incomplete radiofrequency ablation (iRFA) are unclear. This study aimed to provide further insight into the integrated transcriptomic and proteogenomic landscape and identify a new target involved in HCC progression following iRFA.

**Methods:**

Peripheral blood and matched tissue samples were collected from 10 RFA-treated HCC patients. Multiplex immunostaining and flow cytometry were used to assess local and systemic immune responses. Differentially expressed genes (DEGs) and differentially expressed proteins (DEPs) were explored via transcriptomic and proteogenomic analyses. Proteinase-3 (PRTN3) was identified in these analyses. And then, the ability of PRTN3 to predict overall survival (OS) was assessed in 70 HCC patients with early recurrence after RFA. In vitro CCK-8, wound healing and transwell assays were conducted to observe interactions between Kupffer cells (KCs) and HCC cells induced by PRTN3. The protein levels of multiple oncogenic factors and signaling pathway components were detected by western blotting. A xenograft mouse model was built to observe the tumorigenic effect of PRTN3 overexpression on HCC.

**Results:**

Multiplex immunostaining revealed no immediate significant change in local immune cell counts in periablational tumor tissues after 30 min of iRFA. Flow cytometry showed significantly increased levels of CD4^+^ T cells, CD4^+^CD8^+^ T cells, and CD4^+^CD25^+^CD127^−^ Tregs and significantly decreased the levels of CD16^+^CD56^+^ natural killer cells on day 5 after cRFA (*p* < 0.05). Transcriptomics and proteomics revealed 389 DEGs and 20 DEPs. Pathway analysis showed that the DEP-DEGs were mainly enriched in the immunoinflammatory response, cancer progression and metabolic processes. Among the DEP-DEGs, PRTN3 was persistently upregulated and closely associated with the OS of patients with early recurrent HCC following RFA. PRTN3 expressed in KCs may affect the migration and invasion of heat stress-treated HCC cells. PRTN3 promotes tumor growth via multiple oncogenic factors and the PI3K/AKT and P38/ERK signaling pathways.

**Conclusions:**

This study provides a comprehensive overview of the immune response and transcriptomic and proteogenomic landscapes of the HCC milieu induced by iRFA, revealing that PRTN3 promotes HCC progression after iRFA.

**Trial registration:**

ChiCTR2200055606, http://www.chictr.org.cn/showproj.aspx?proj=32588.

**Supplementary Information:**

The online version contains supplementary material available at 10.1186/s13046-023-02716-y.

## Introduction

Hepatocellular carcinoma (HCC) remains a health challenge worldwide; its incidence is increasing, and it accounts for ~ 90% of liver cancers [1–3]. Surgery, liver transplantation and ablation are potentially curative options but are only amenable for early-stage patients. Additionally, the high rate of tumor recurrence limits long-term survival [4]. In advanced-stage HCC, oral multityrosine kinase inhibitors (e.g., sorafenib and lenvatinib in the first-line setting; regorafenib and cabozantinib in the second-line setting) could prolong the survival of patients, but their benefits are negligible [5, 6]. Although HCC is an attractive target for immunotherapy, various randomized trials of monotherapy in recent decades have not yielded a satisfactory objective response rate and significant improvement in overall survival (OS) [7]. Therefore, the development of combination strategies for this deadly disease has led to novel avenues to improve therapeutic efficacy.

Radiofrequency ablation (RFA) is a common therapeutic technique to destroy HCC through a Joule effect induced by the generation of high frequency alternating current and local heat from an electrode tip inserted into neoplastic tissues [8]. Especially in very-early-stage HCC, complete RFA (cRFA) is considered a standard treatment option as a minimally invasive locoregional therapy (LRT) with lower morbidity rates and cost than other options [9]. However, RFA is not favored in cases with tumors larger than 2 cm, as this group has a lower rate of complete response and a higher recurrence rate than the group with smaller tumors [10]. Multiple studies have demonstrated that several clinical characteristics of tumor-like size, poorly defined HCC margins, and location of the tumor near the portal vein branches, potentially resulting in incomplete RFA (iRFA), are significant risk factors for early local recurrence (< 2 years) of HCC [11, 12]. Moreover, two retrospective cohort studies [13, 14] also confirmed that iRFA was significantly related to distant recurrence as well as local recurrence. To date, the mechanisms of early relapse after iRFA remain to be fully understood. Several studies provide potential explanations for this phenomenon. First, rapid heating-based RFA may lead to an unpredicted increase in internal pressure in tumors and subsequently cause malignant cell dislodging and scattering to adjacent sites [15, 16]. Second, RFA could induce the formation of an inflamed periablational “red zone”, in which elevation of IL-6, vascular endothelial growth factor (VEGF) and hepatocyte growth factor (HGF) induce a chronic inflammatory condition that supports the formation of a protumorigenic microenvironment in the liver [17]. Third, RFA inciting liver regeneration may simultaneously exacerbate such undesired outcomes in response to the release of tumor-promoting cytokines and growth factors [18].

It is worth mentioning that LRTs in HCC, including ablation, transarterial (chemo)embolization TA(C)E, hepatic artery infusion chemotherapy (HAIC) and yttrium 90 radioembolization, can induce a peripheral immune response through induction of an inflammatory response and tumor-associated antigen release [19]. Following RFA, a murine model showed that the infiltration of dendritic cells (DCs) and CD4^+^ and CD8^+^ T-cell responses were significantly increased in tumors [20]. Inconsistently, RFA-treated mice displayed a transient antitumor effector function of CD4^+^ and CD8^+^ T cells and were quickly tamed by active immune suppression responses, driving a shift to a higher regulatory T-cell to CD8^+^ cell ratio and PD-L1/PD-1 expression [21]. A clinical observation of RFA-treated HCC patients revealed that RFA could enhance tumor-associated antigen-specific T-cell responses. However, their memory phenotype and survival times are not sufficient to prevent HCC recurrence [22]. Fortunately, concomitant treatment with immunotherapy and RFA therapy could synergistically enhance antitumor immunity. For example, combination therapy with RFA plus immune checkpoint inhibitors such as anti-PD-1 [23] and cytotoxic T-lymphocyte-associated protein 4 (CTLA-4) [24] was superior to RFA alone in prolonging the survival of HCC patients. Of note, even if RFA combined with immunotherapy emerged as an encouraging antitumor strategy for HCC, iRFA could limit the effect of immunotherapy [25].

Currently, the in-depth mechanisms of early recurrence of HCC and various cell immune responses initiated by RFA therapy remain to be further clarified. Animal models may address these of the key barriers to human studies. However, most human data on immune responses induced by RFA were derived from the exploration of peripheral blood samples. Comprehensive data analysis of human tumor tissues is likely important for the translation of RFA treatment in HCC. Hence, we designed a novel prospective clinical trial to directly obtain unablated HCC tissue adjacent to RFA-treated tissue (1 cm away) via a biopsy needle during surgery following the implementation of cRFA. Modulation of molecules and signaling pathways in the tumor microenvironment (TME) induced by iRFA was comprehensively dissected by integrating the transcriptome and proteome. This study may provide new insights into the mechanism underlying the impacts of iRFA on tumor progression and the cell immune response and contribute to the development of combinatorial immunotherapeutic strategies to decrease the recurrence rate of HCC.

## Materials and methods

### Clinical sample acquisition

**Fig. 1:**
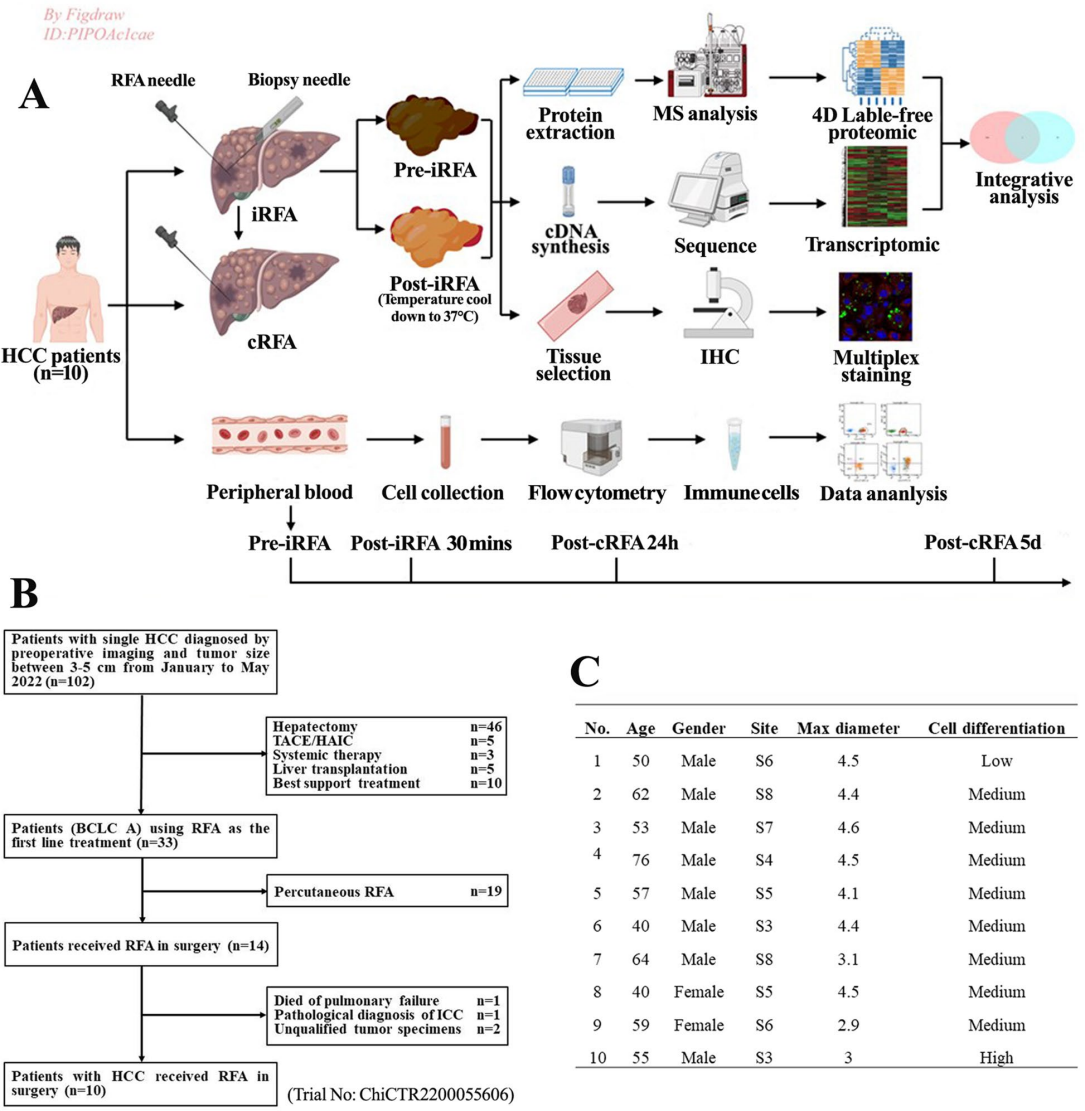
Comprehensive exploration of the immune response, transcriptomics and proteomics landscapes in HCC patients after iRFA. **A** Scheme of experiments conducted on each sample from 102 HCCs. **B** The flow chart of patient selection. **C** The baseline characteristics of 10 HCC patients

One cohort of 102 consecutive HCC patients recruited from January to May 2022 and diagnosed by imaging with tumor sizes between 3–5 cm were initially evaluated in this prospective observational study. As shown in Fig. 1, ten patients who satisfied the eligibility criteria received RFA treatments during surgery and provided peripheral blood and matched tissue samples for flow cytometry, multiplex immunofluorescence staining, and transcriptomic and proteogenomic analyses. Another cohort of 70 patients who underwent a second RFA procedure between January 2016 and December 2018 due to early recurrence after the first RFA procedure in the retrospective study provided samples for tissue microarrays. All patients in both cohorts ultimately recruited for this study were pathologically diagnosed with primary HCC. In addition, none of the patients had preoperative extrahepatic metastases or received anticancer treatments before the operation. This study was performed in compliance with the 1975 Helsinki Declaration and was approved by the Ethics Review Committee of the First Affiliated Hospital of Chongqing Medical University (No. 2021–668). This prospective observational study followed the STROBE guidelines. Informed consent to participate in this study was obtained from the research subjects.

### Experimental design

Ten patients who satisfied the eligibility criteria (BCLC 0-A) were enrolled in this prospective study of iRFA treatment at a 1 cm ablating range for 5 min. This ablation system consisted of a 105-Watt generator and a length-adjustable 16-gauge single electrode (LDRF-120S, Lead Electron Corporation, Mianyang, China). While the temperature of the tip cooled below 37 °C for 30 min, periablational HCC tissues adjacent to the iRFA tumor margin (1 cm) were obtained via a 16-gauge biopsy needle, and then cRFA was performed for 15 min. To ensure the accuracy of RFA and acquisition of tumor tissues, all target tumors were required to be between 3–5 cm in diameter and from within 1 cm of the liver surface. All tumors were treated in situ after ultrasound-guided placement of the needle electrode. Before RFA, both tumor tissues and peripheral serum samples (as controls) were collected, and serum samples were also obtained at 30 min post-iRNA, 24 h post-cRFA and 5 days post-cRFA. Flow cytometry and multiplex immunofluorescence staining were used to detect the immune cell response after iRFA/cRFA. A portion of the fresh tissue sample was immediately placed in liquid nitrogen and then transferred to -80 °C within 30 min for subsequent transcriptomic and proteomic analyses (Fig. 1A).

### Cell line

The human HCC cell lines Hep3B and SMMC-7721 (Academy of Life Sciences of Chongqing Medical University) were cultured in Dulbecco's modified Eagle's medium (DMEM) supplemented with 10% fetal bovine serum and 1% penicillin/streptomycin. To establish an in vitro sublethal heat stress model, HCC cells (SMMC-7721 and Hep3B) were cultured with medium preheated at 47 °C in a water bath apparatus for 15 min. After heat exposure, the cells were cultured for 1 h and then used for further experiments.

### Flow cytometry

Flow cytometry was performed according to the standard protocol as previously described [26]. The primary antibodies used are listed in Table 1.

**Table 1:** Antibodies for flow cytometry, immunofluorescence and western blotting

Antibody	Source	Application	Catalogue No
CD3	BD Biosciences	FC	555,339
CD4 (PE-Cy7)	BD Biosciences	FC	557,852
CD4 (PerCP-Cy5.5)	BD Biosciences	FC	566,923
CD8 (APC-Cy7)	BD Biosciences	FC	348,793
CD16 + CD56 (PE)	BD Biosciences	FC	340,500
CD19 (APC)	BD Biosciences	FC	555,415
CD25 (PE)	BD Biosciences	FC	555,432
CD45 (PerCP-Cy5.5)	BD Biosciences	FC	340,952
CD45 (APC-Cy7)	BD Biosciences	FC	557,833
CD127 (APC)	eBioscience	FC	17–1278-42
CD4	eBioscience	IF	14–2444-82
CD8	DAKO	IF	ab17147
CD19	DAKO	IF	ab270715
CD25	Invitrogen	IF	24,204
CD56	Abcam	IF	ab6123
CD127	Thermo Fisher	IF	605–210
CD68	Abcam	IF	ab955
PRTN3	ZEN BIO	IHC	389,254
CXCL5	Abcam	WB	ab305100
MPO	Abcam	WB	ab208670
MMP9	Abcam	WB	ab76003
IL-6	Abcam	WB	**ab233706**
p-AKT	Cell signaling	WB	9271
p-ERK1/2	Cell signaling	WB	4370
p-P38	Cell signaling	WB	4511
PI3K	Cell signaling	WB	4249
GAPDH	Abcam	WB	Ab8245

*Abbreviations*: *FC* Flow cytometry, *IF* Immunofluorescence, *IHC* Immunohistochemistry, *WB* Western blotting

### Multiplexed immunofluorescence

Multiplex immunofluorescence staining was performed as previously described [27]. Tyramide signal amplification (Opal 7-color Manual IHC Kit, NEL801001KT; PerkinElmer) was used to assess distinct fluorophore binding, and the antibodies were applied in the following order: anti-CD3/Opal 650, anti-CD4/Opal-620/Opal-520, anti-CD8/Opal-570, anti-CD19/Opal-690, CD25/Opal-570, CD56/Opal520, and anti-CD127/Opal-620. Then, the sections were counterstained with DAPI (Sigma‒Aldrich), mounted with VECTASHIELD® HardSet Antifade Mounting Medium (H-1400, Vector Labs) and imaged by the Automated Quantitative Pathology Imaging and Analysis platform (Vectra Polaris, PerkinElmer).

### RNA-seq analysis and data processing

Total RNA was isolated from the tissues using TRIzol reagent (Invitrogen) according to the manufacturer’s instructions. A total amount of 1 μg RNA per sample was used for analysis. The libraries were sequenced using the UltraTM RNA Library Prep Kit (Illumina, New England Biolabs) according to the manufacturer’s guidelines, and index codes were added to attribute sequences to each sample. After size selection using Agencourt AMPure XP (Beckman Coulter, Beverly, USA), PCR products were purified (AMPure XP system), and library quality was assessed on the Agilent Bioanalyzer 2100 system. After cluster generation on a cBot Cluster Generation System (TruSeq PE Cluster Kit v3-cBot-HS, Illumina), 150 bp paired-end reads were generated on an Illumina NovaSeq platform. Paired-end clean reads were aligned simultaneously to the reference genome using HISAT2 v2.0.5. The mapped reads of each sample were assembled by StringTie (v1.3.3b) in a reference-based approach for novel transcript prediction. The DESeq2 R package (1.16.1), which employs a model based on the negative binomial distribution, was used to analyze the DEGs of the two groups. The resulting *p* values were adjusted using Benjamini and Hochberg’s approach for controlling the false discovery rate. Genes with an adjusted *p* value < 0.05 found by DESeq2 were considered differentially expressed. Gene Ontology (GO) and Kyoto Encyclopedia of Genes and Genomes (KEGG) analyses of DEGs were implemented via the clusterProfiler R package. The weighted correlation network analysis (WGCNA) R package was used to describe the gene association modes among different samples. Protein‒protein interaction (PPI) analysis of DEGs was based on the STRING database (https://cn.string-db.org/). GATK2 (v3.7) and rMATS (3.2.5) software were used to perform SNP calling and alternative splicing analysis, respectively.

### Proteogenomic analysis

The MS data were analyzed using the label-free strategy of MaxQuant software (1.6.17.0) and searched against the UniProt_*HomoSapiens*_20387_20210928_9606_swissprot database. An initial search was set at a precursor mass window of 10 ppm. The search followed an enzymatic cleavage rule of Trypsin/P and allowed a maximum of two missed cleavage sites and a mass tolerance of 40 ppm for fragment ions. Carbamidomethylation of cysteines was defined as a fixed modification, while protein N-terminal acetylation and methionine oxidation were defined as variable modifications for database searching. The cutoff of the global false discovery rate (FDR) for peptide and protein identification was set to 0.01. Protein abundance was calculated based on the normalized spectral protein intensity (LFQ intensity). Proteins with a fold change > 2 or < 0.5 and *p* value (Student’s t test) < 0.05 were considered to be differentially expressed proteins.

### Integrative proteomic and transcriptomic analysis

The correlations for the expression of a gene and its corresponding protein were compared between stages. Venn diagrams were constructed to reveal the factors that were dysregulated at both the gene and protein levels. Correlations were analyzed according to the Pearson correlation coefficient for each pair of pre- and postiRFA tumor tissues. The four-quadrant graph shows a correlation analysis between the proteome and transcriptome levels. GO and KEGG functional enrichment analyses were also conducted for relevance analysis.

### Bioinformatics analyses of PRTN3

The coexpression networks of PRTN3 in HCC tissues and adjacent liver tissues were explored via the Hepatocellular Carcinoma Database (HCCDB). Simultaneously, the Search Tool for the Retrieval of Interacting Genes (STRING, https://cn.string-db.org/) was applied to identify functional interactions between PRTN3 and the top 10 proteins with the strongest associations in HCC. The Tumor Immune Estimation Resource (TIMER, https://cistrome.shinyapps.io/timer/) was used to investigate the relationship between six tumor-infiltrating immune subsets and PRTN3 expression in HCC, and the gene module and somatic copy number alteration (SCNA) module were chosen for subsequent analysis.

### Tissue microarray (TMA) and IHC analysis

A TMA was constructed as described previously [28, 29]. IHC was performed following the manufacturer’s instructions (Invitrogen, Zymed Polymer Detection System). The four most representative areas were observed (at 400X magnification) after positively stained cells were screened (at 100X magnification) under a Leica DMLA light microscope (Leica Microsystems, Wetzlar, Germany).

### Isolation of human Kupffer cells

Normal liver tissues of hepatic hemangiomas were used to isolate primary liver nonparenchymal cells with gradient density centrifugation by Nycodenz gradients (Axis Shield, Rodelokka, Norway) as described previously [30]. Briefly, liver tissues were cut into 0.5–1.0 cm thick slices and digested for 30 min at 37 °C with pronase (Roche), collagenase (Gibco, Invitrogen corporation) and DNase (Roche). Kupffer cells (KCs) were separated from other nonparenchymal cells by 16.7% Nycodenz solutions after filtration and centrifugation. The mixtures were centrifuged without braking, and the interfaces were then collected. Then, the cell suspension was added to cell culture plates and incubated. KCs adhered during the incubation time and were collected and cultured at 37 °C in 5% CO2 in DMEM with fetal bovine serum. Immunofluorescence staining of cells with CD68 further confirmed the extracted KCs [31, 32].

### Cell transfection of lentiviral vectors

A lentiviral vector for PRTN3 overexpression (PR3OE) or a lentiviral vector containing shRNA-PRTN3 to knockdown PRTN3 (PR3KD) were synthesized (Shanghai Genechem Co. Ltd., Shanghai, China) and used to transfect KCs and the two HCC cell lines. Stable transfectants were characterized by quantitative real-time polymerase chain reaction (qRT–PCR).

### Real-time quantitative PCR

As described previously [28], total RNA was extracted from cells using TRIzol Reagent (Life Technologies, Carlsbad, CA, USA) according to the manufacturer's instructions. The assay ID was PRTN3 (Hs01597751_g1). The level of the target gene was normalized to that of β-actin (Hs01060665_g1).

### Collection of KC-conditioned medium

After 24 h of culture with lentiviral transfectants, KCs were washed twice with serum-free DMEM and incubated for 24 h with serum-free DMEM. KC-conditioned medium (CM) was then collected as described previously [30].

### Cell proliferation assay

For cell growth assays, a total of 5 × 10^3^ sublethal heat stress-treated HCC cells cultured with KC-CM for 24 h were maintained in 96-well plates. CCK-8 solution (Dojindo, Japan) was used to measure cell viability at 0, 24, 48 and 72 h.

### Wound healing assay

Sublethal heat stress-treated HCC cells cultured with KC-CM for 24 h were seeded and cultured in 6-well plates (1 × 10^6^/well) to 90% confluence. Cell monolayers were scratched with a sterile 200-μl pipette tip to form a wound/gap, and then the relative wound area change was observed at 0, 24 and 48 h by inversion microscopy (Olympus, Japan).

### Cell migration and invasion assays

As described previously [26], 1 × 10^5^/well of sublethal heat stress-treated HCC cells cultured with KC-CM for 24 h were placed in an 8-μm pore size upper chamber (24-well insert, Millipore, USA) with or without a Matrigel (BD, USA). Complete medium was added to the lower chambers. After 24 h of incubation, cells were fixed with methanol and stained with crystal violet.

### Western blotting

Western blotting was performed according to the standard protocol. The proteins were then detected using ECL reagent (Millipore, Billerica, MA, USA). The primary antibodies used are listed in Table 1. After sublethal heat stress treatment, the proteins of HCC cells were detected.

### Xenograft model

To study the direct tumor-promoting role of RFA on tumor cells via PRTN3, sublethal heat stress-treated HCC cells (SMMC-7721 and Hep3B) with or without PR3OE transfection were resuspended in 100 μL phosphate-buffered saline (PBS) containing 1 × 10^7^ HCC cells and injected subcutaneously into 6-week-old Balb/c-nude mice. Tumor volume was measured once every 3 days and calculated as volume = (width^2^ × length)/2. All mice were sacrificed on day 35. All animal care and experimental protocols were performed in line with the guidelines of the Animal Ethics Committee of Chongqing Medical University.

### Statistical analysis

The *χ*^2^ test or Fisher’s exact test was used to compare the categorical variables, including the differences in demographic and clinical characteristics. Two-sided Student’s t tests with a normal distribution or nonparametric Mann–Whitney U tests with an irregular distribution were used to compare the differences between two groups and are reported as the mean ± standard deviation. One-way ANOVA was used to compare the differences among three or four groups. Pearson’s or Spearman’s ρ coefficient tests were used to evaluate the correlation matrices. The medians were used as cutoff values of continuous variables in patients’ characteristic variables. OS was defined as the interval between the date of first RFA and death or the last follow-up. Survival curves were generated using Kaplan‒Meier estimates and compared using the log-rank test. The “minimum *p* value” approach [28] was used to obtain an optimal cutoff for the best separation according to OS. *p* < 0.05 was considered statistically significant. Statistical analyses were performed using SPSS 24.0 (SPSS, Inc., Chicago, IL, USA).

## Results

### Selected patients

The flow chart of the study is presented in Fig. 1B. The patients were 40–76 years old. There were 2 female patients and 8 male patients with tumor diameters of 2.9–4.6 cm. Eight patients had moderately differentiated tumors, one patient had a poorly differentiated tumor, and one patient had a well-differentiated tumor (Fig. 1C). Complete ablation was achieved after surgery, and no serious complications occurred in any of the patients.

### Immune cells in peripheral blood and infiltration in tumors in response to RFA

**Fig. 2:**
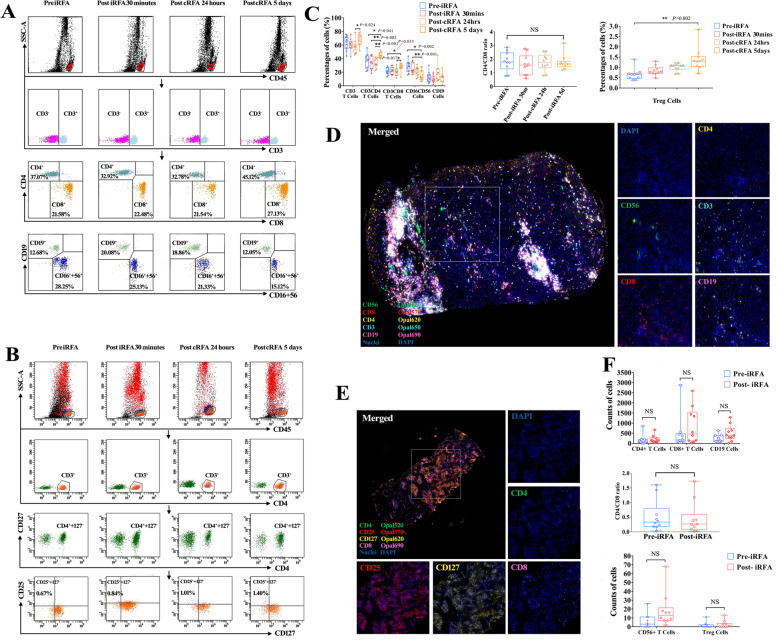
Immune response of HCC patients after RFA. **A** Representative flow cytometric plots showing the percentages of CD4^+^, CD8^+^, CD19^+^, and CD16^+^ + CD56^+^ cells before RFA, 30 min after iRFA, and 24 h and 5 days after cRFA. **B** Representative flow cytometric plots showing the percentages of CD4^+^ + CD25^+^ + CD127^−^ Treg cells before RFA, 30 min after iRFA, and 24 h and 5 days after cRFA. **C** Quantitative assessment of flow cytometric results. **D** Representative images of multiplex staining of CD4^+^, CD8^+^, CD19^+^, CD16^+^ and CD56^+^ cells in HCC samples after iRFA. **E** Representative images of multiplex staining of CD4^+^, CD25^+^, and CD127^+^ cells in HCC samples after iRFA. **F** Quantitative assessment of the multiplex staining results

Systemic and local immune responses were observed in peripheral blood by flow cytometry (Fig. 2A-C) and HCC tissues by multiplexed immunofluorescence analyses (Fig. 2D-F), respectively. Consistent with the change in circulating immune cell counts, there was no significant change in immune cell count or the CD4^+^/CD8^+^ ratio in periablational tumor tissues after 30 min of treatment with iRFA (Fig. 2C and F, *p* > 0.05). However, the counts of CD4^+^ (*p* = 0.041) and CD8^+^ (*p* = 0.033) T cells and CD4^+^CD25^+^CD127^−^ Tregs (*p* = 0.002) significantly increased on day 5 following cRFA (with before RFA counts as the baseline). Conversely, CD16^+^CD56^+^ NK cells showed a significant decrease on day 5 following cRFA (*p* = 0.002). There were no significant changes in CD19^+^ cell counts or the CD4^+^/CD8^+^ ratio during the whole trial. Compared to day 5, some immune cells, such as CD3^+^ cells (*p* = 0.024), CD4^+^ (*p* < 0.001) and CD8^+^ (*p* = 0.017) T cells, began to present an upward trend 24 h after ablation therapy. We also found that there were increased numbers of CD4^+^ T cells and decreased numbers of CD16^+^CD56^+^ NK cells on day 5 compared with 24 h following RFA treatment (Fig. 2C). All these dramatic changes suggested that RFA stimulation did not provoke immediate local and systemic immune responses and instead initiated a gradual cascade response in the systemic immune milieu, most likely initiated by local effects.

### DEGs in HCC after iRFA

**Fig. 3:**
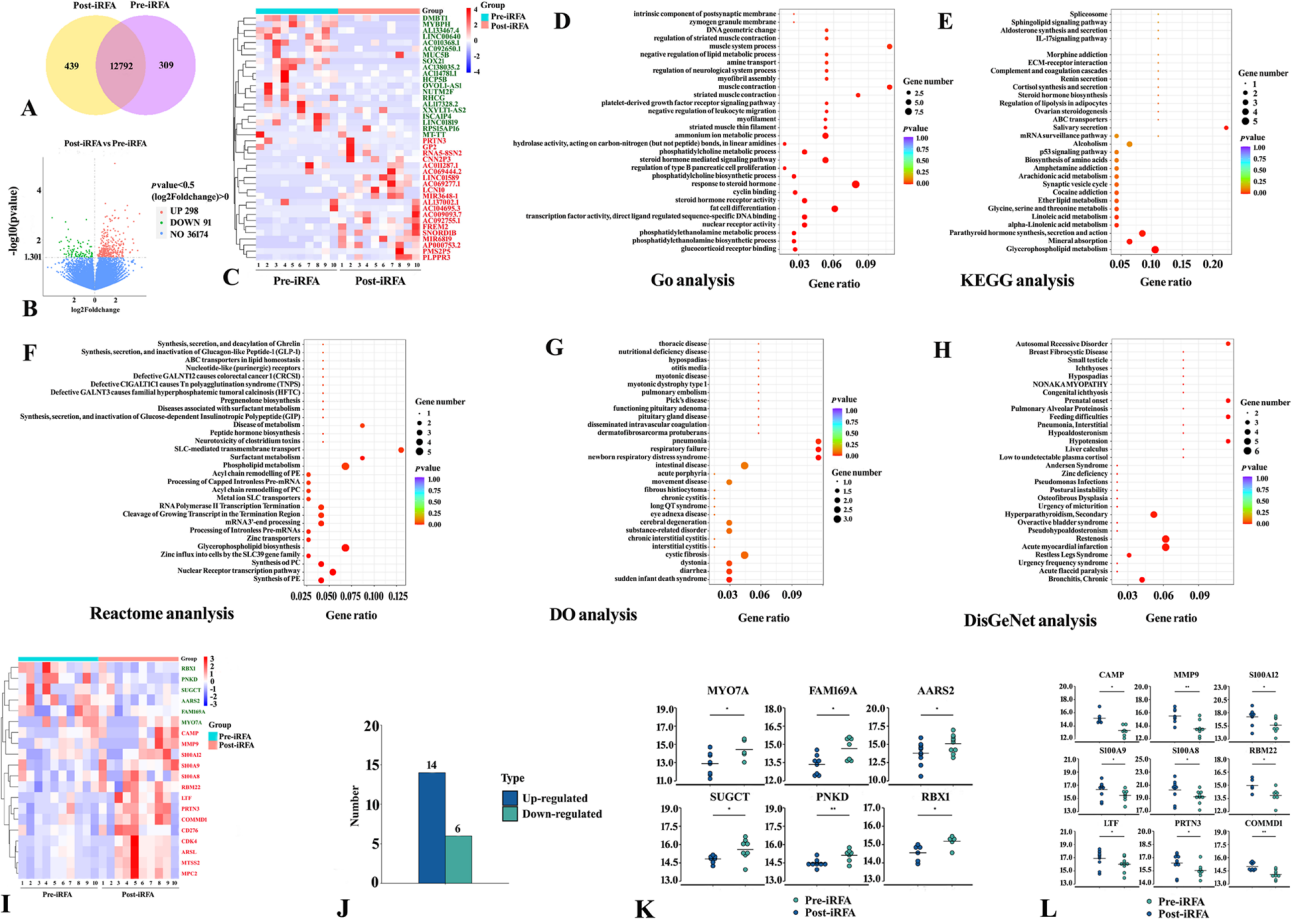
Transcriptomics and proteomics analysis. **A** Venn diagrams showing the overlap of detected genes in HCC tissues between pre-iRFA and post-iRFA. **B** and **C** Volcano plots and heatmaps showing the differentially expressed transcripts. **D**-**H** GO, KEGG, Reactome, DO and DisGeNet enrichment analysis of the top 30 transcriptomic datasets. **I** Heatmaps showing the DEPs. **J** Numbers of up- and downregulated DEPs. **K** Quantitative results for 6 downregulated DEPs. **L** Quantitative results for the top 9 downregulated DEPs

Sequencing fragments from all samples were converted into sequence data (reads) and subjected to data quality control, including error rate distribution assessment, GC content distribution assessment and sequencing data filtering (Figure S1). Finally, reliable clean reads were obtained. Moreover, the FeatureCounts tool in Subread software was used to align reads from multiple regions of the genome to complete the quantification and distribution analysis of DEGs and the sample correlation analysis (Figure S2). A total of 13,540 genes were identified after calculating the expression level of each mapped gene (Fig. 3A), and the number of altered genes increased after iRFA (pre-iRFA vs. post-iRFA: 309 vs. 439). Among all DEGs (*n* = 389), 298 were upregulated and 91 were downregulated in HCC tissues following iRFA, as shown in the volcano plot (Fig. 3B and Table S1), and the top 20 downregulated and upregulated DEGs are shown in Fig. 3C. Next, we performed a functional analysis associating DEGs with GO categories, covering three domains: biological process (BP), cellular component (CC) and molecular function (MF). Some key functions, such as the PDGFR signaling pathway (BP), metabolic process (e.g., BP: phosphatidylethanolamine, phosphatidylcholine, ammonium ion), biosynthetic process (e.g., BP: phosphatidylethanolamine, cellular amino acid), nuclear speck (CC), nuclear receptor activity (MF), transcription factor activity (MF), and binding function (e.g., MF: cyclin binding, glycine binding, pyridoxal phosphate binding, RNA polymerase binding, RNA polymerase II core binding), were greatly affected by iRFA (Fig. 3D, *p* < 0.05). To further understand the biological processes of iRFA-disturbed HCC, KEGG pathway analysis was performed. Similarly, modulations of multiple metabolic pathways, such as those involving glycerophospholipids, alpha-linolenic acid, linoleic acid, ether lipids, glycine, serine and threonine, occurred in HCC microenvironments. Notably, MUC5B, CXCL2 and FOSB were the DEGs involved in the IL-17 signaling pathway (Fig. 3E). As shown in Fig. 3F-H, we also found various regulation of biological response genes, disease-related genes and their functions in Reactome, DisGeNET and GO enrichment analyses. Referring to the Cosmic database, another important finding was that iRFA induced the upregulation of many potential proto-oncogenes, such as PRTN3, PIDD1, KLRC2, CDK3, CXCL2, and MMP19, and the downregulation of some tumor suppressor genes, such as DMBT1, RHCG, and PDGFRL ZRANB3. These data indicated the potential molecular mechanisms associated with tumor progression.

### Alternative splicing, single nucleotide polymorphisms, and fusion events

To further explore the effect of iRFA on genetic mutation, we analyzed alternative splicing, single nucleotide polymorphisms, and fusion events at the transcriptomic level. The alternative splicing events were investigated by rMATS software. We detected differential alternative 3′ splice site (A3SS) events in UBXN11 and skipped exon (SE) events in LRR1, IQCB1, ZNF26, AHI1, KIAA1191, ENTR1 and LCN12, but mutually exclusive exons (MXEs), alternative 5' splice sites (A5SSs), and retained introns (RIs) were not detected (Figure S3). The exon inclusion levels of these DEGs were significantly decreased in HCC tissues after iRFA. The results further demonstrated the stimulatory effect of iRFA in the TME by modulating RNA splicing. Furthermore, the frequency of insertion‒deletion (INDEL) and single nucleotide polymorphism (SNP) alterations in various genes was analyzed by GATK and SnpEff, revealing the prevalent variations in the function, region and impact of INDELs and SNPs (Figure S4). With the RNA-seq data, we searched for gene fusions and found PTCHD4/DDAH1 (5/10 paired HCC samples, 50%) as the most recurrent gene fusion (Figure S5). ANO1-related fusion events were identified in 30% of paired HCC samples. These fusion gene events on chromosomes may infer an important role for iRFA in the promotion of HCC progression.

### Proteogenomic landscape of HCC after iRFA

**Fig. 4:**
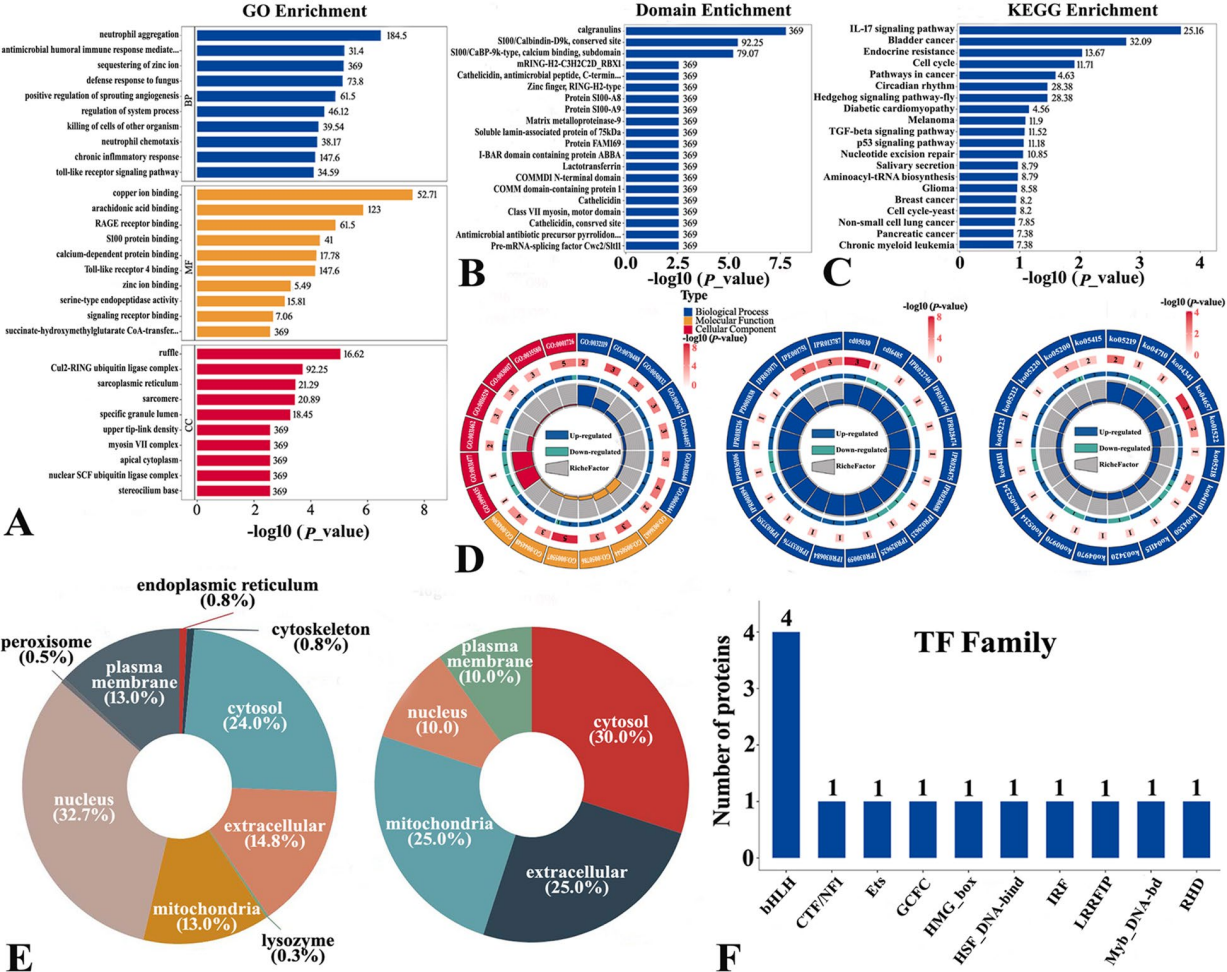
Enrichment analysis, altered subcellular localization and transcription factor analysis of DEPs. **A**-**C** GO, domain and KEGG enrichment analyses of the proteomics data. **D** Circus plot of enrichment analysis showing differences in proteomics profiles. Top 20 GO terms, pathways and domains in the outer track. The numbers and -log10 (*p* value) of DEPs in the second outer tracks: the length of each band represents the number of DEPs. The *P* value (smaller) corresponds to color intensity (redder). The third tracks show the ratio of DEPs. Blue and cyan represent the proportion of up- and downregulated proteins, respectively. The gridlines in the fourth tracks indicate the RichFactor values. **E** Subcellular localization of DEPs before iRFA and after iRFA. F: Protein number of the top 10 transcription factor families

The reproducibility of protein quantification was evaluated by relative standard deviation and Pearson's correlation coefficient, and a total of 72,533 unique peptides and 7380 proteins were quantified (Figure S6). As shown in Fig. 3I-L, 20 proteins were defined as DEPs after iRFA stimulation, among which 14 were upregulated and 6 were downregulated (Table S2). Notably, we detected several critical regulating proteins associated with cancer processes, such as CAMP, MMP9, the S100 family (100A8, S100A9, S100A12), and PRTN3, among the proteins with the most significant increases (Fig. 3L). To further understand the proteomic alterations produced following iRFA, enrichment analyses were carried out on our proteomic data. The clustering of DEPs combined with enrichment analyses showed marked changes in genes involved in cancer process regulation **(**Fig. 4A-D). In particular, the enriched GO terms indicated that inflammatory/immune responses, such as neutrophil aggregation and chemotaxis, antimicrobial humoral immune response mediated by antimicrobial peptide, killing of cells, Toll-like receptor signaling pathway, positive regulation of inflammatory response, and S100 protein binding, were triggered by iRFA. KEGG pathway enrichment analysis at the protein level revealed marked enrichment of cancer progression- and inflammation/immune-related pathways, such as the IL-17 signaling pathway, cell cycle, pathways in cancer, Hedgehog signaling pathway, TGF-beta signaling pathway, and p53 signaling pathway, with iRFA treatment. In terms of protein function, some domains of proteins also changed significantly. Furthermore, iRFA altered the subcellular localization of DEPs (Fig. 4E and Table S3), as more DEPs were found in the mitochondria (25%) and the extracellular space (25%) after iRFA (pre-iRFA: 13% and 14.8%, respectively). The subcellular relocalization of DEPs suggested that iRFA could be associated with increased mitochondrial biogenesis and remodeled extracellular function, which could provide cells with higher energy production capability upon proliferation of tumor cells (mitochondrial) and cellular communication (extracellular), including interactions between HCC cells and distinct immune cells. Based on the AnimalTFDB and PlantTFDB databases, the DEPs were members of fourteen transcription factor families (Fig. 4F and Table S4), some of which were previously demonstrated to participate actively in the progression of HCC (e.g., bHLH, ETs, HMG_box, IRF, Myb, and HSF).

### Transcriptomics and proteomics integration

**Fig. 5:**
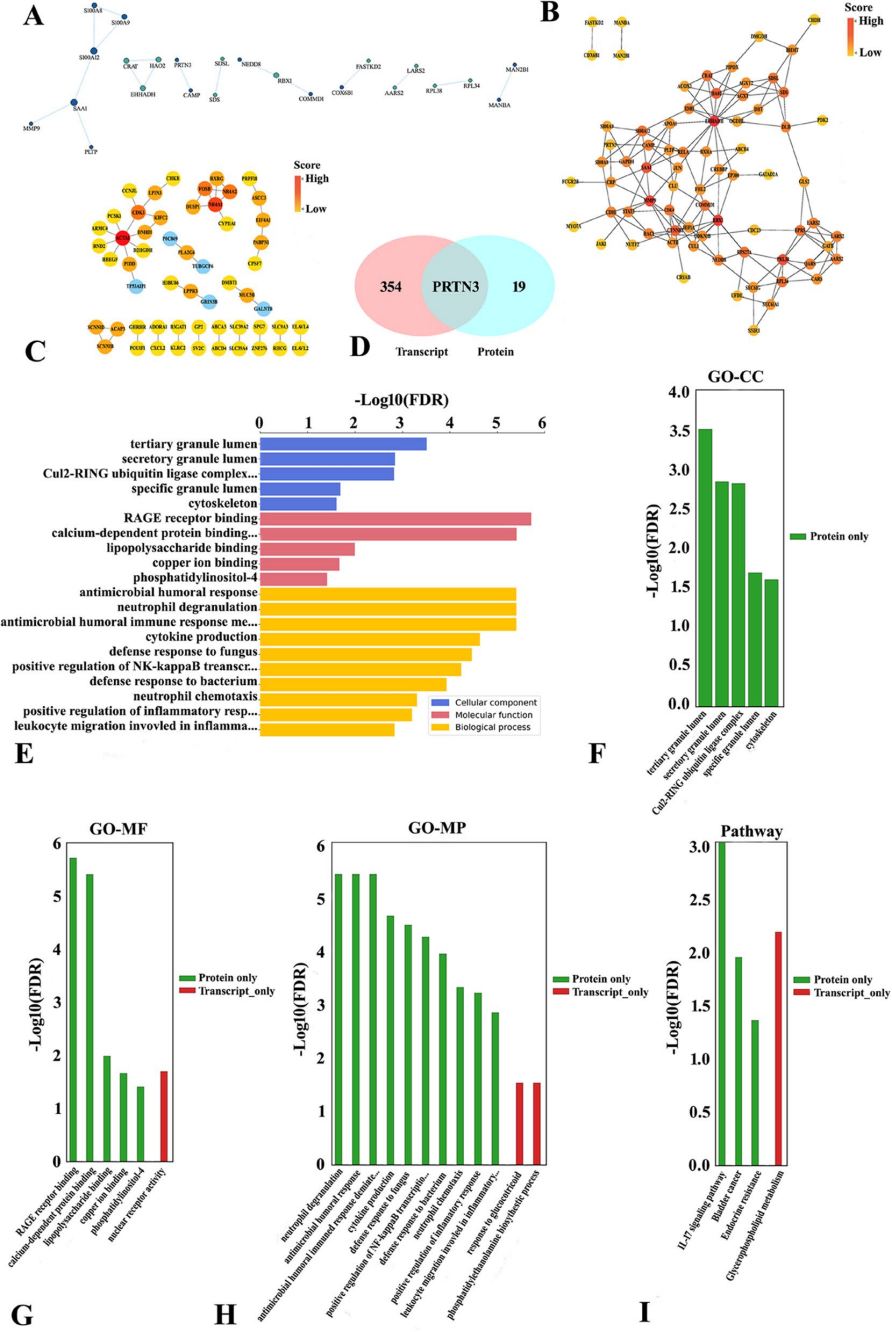
Integrated transcriptomics and proteomics analyses. **A** Phylogenic tree showing PPI based on the top 50 up- and downregulated proteins in proteomics. The size of the circle represents the degree value of protein interaction. Blue and cyan represent the up- and downregulated proteins, respectively. **B** PPI diagram based on the top 100 up- and downregulated proteins in proteomics. **C** PPI diagram based all DEPs in transcriptomics. **D** Venn diagrams showing that only PRTN3 was upregulated in both transcriptomics and proteomics. **E** Integrated transcriptomics and proteomics analyses showing significantly different GO terms only in proteomics. **F**-**I** GO and KEGG enrichment analyses of integrated transcriptomics and proteomics analyses showing different GO terms and KEGG pathways, respectively

Based on the STRING database, we compared the PPIs of DEGs and DEPs and visualized protein regulatory networks using Cytoscape software (Fig. 5A-C). PPI networks based on transcriptomics analysis revealed that SAA1, MMP9, EHHADH, and RBX1 may be important regulatory proteins in the networks (Fig. 5B). Derived from all DEGs of proteomics data, the PPI networks indicated that ACTA1 and NR4A1 were the pivotal genes in the networks (Fig. 5C). The Venn diagram of the integrated analysis showed that PRTN3 was the only overlapping gene/protein upregulated at both the transcriptomic and proteomic levels (Fig. 5D), suggesting the existence of posttranscriptional regulation and potentially affecting the HCC process of PRTN3 after iRFA. Furthermore, we integrated the transcriptome and proteome data for GO and KEGG pathway enrichment analyses to identify the dysregulated genes/proteins with variations (Fig. 5E-I). GO enrichment analysis at the two omics levels showed that there were more enriched GO terms at the protein level than at the transcript level (Table S5). For example, some important tumor growth- and immune/inflammatory response-associated GO enrichment analysis results at the protein level were as follows: Cul2-RING ubiquitin ligase complex (CC), cytoskeleton (CC), RAGE receptor binding (MF), calcium-dependent protein binding (MF), lipopolysaccharide binding (MF), copper ion binding (MF), cytokine production (BP), positive regulation of NF-kappaB transcription factor activity (BP), neutrophil chemotaxis (BP), and positive regulation of inflammatory response (BP). KEGG pathway analysis suggested significant variations in the IL-17 signaling pathway at the protein level. This difference between the transcriptome and proteome might be related to the fact that mRNA data do not accurately reflect posttranslational processes, and protein regulatory processes could operate exclusively at the protein level. Therefore, the correlation analysis methods of the two omics approaches were more suitable for the effective and comprehensive analysis of the immune response and tumor development following iRFA.

### PPI network analysis of PRTN3 and its association with immune infiltration

Given the significantly different expression of PRTN3 in the integrated transcriptomics and proteomics analyses, we used the STRING database to conduct a PPI network analysis and found the strongest interactions with CAMP, IL32, CD177, MPO, SERPINA1, and SERPINB1 (Figure S7A). To further understand the molecular mechanisms of PRTN3 in HCC and adjacent tumor tissues, we used the HCCDB database to conduct a gene expression interaction network analysis (Figures S7B-C) and found that the MPO-PRTN3 signaling pathway was activated in the three PPI network analyses, revealing the potential protumor role of these components as neutrophil protease genes. The inflammatory protein PRTN3 has already been identified as a hub gene in immune-related molecular mechanisms [33] and facilitates immune cell differentiation [34]. Hence, we explored the relationship between the PRTN3 level and immune infiltration in HCC by using the TIMER online tool based on TCGA data (Figure S7D). PRTN3 expression was positively correlated with the infiltration of B cells (*r* = 0.108, *p* = 4.43e-02), CD8^+^ T cells (*r* = 0.138, *p* = 1.09e-09), CD4^+^ T cells (*r* = 0.151, *p* = 4.89e-03), macrophages (*r* = 0.213, *p* = 7.33e-05), neutrophils (*r* = 0.182, *p* = 6.80e-04), and dendritic cells (*r* = 0.125, *p* = 2.15e-02). Multiple genomic aberrations are capable of influencing tumor development and progression through tumor-infiltrating immune cells (TIICs). We also explored the links between genomic aberrations of PRTN3 and the abundance of TIICs through the "SCAN" module in the TIMER database, which provides a comparison of the abundance of TIICs among tumors with different somatic copy number aberrations, including deep deletion, shallow deletion, diploid/normal, low-level gain, and high amplification. We found that the returned box plots showed significantly lower infiltration for most immune cells, except dendritic cells, in tumors with PRTN3 mutations (Figure S7E), indicating an important effect of PRTN3 in the immune response occurring in the inflamed TME.

### PRTN3 is associated with the OS of early recurrent HCC after RFA

**Fig. 6:**
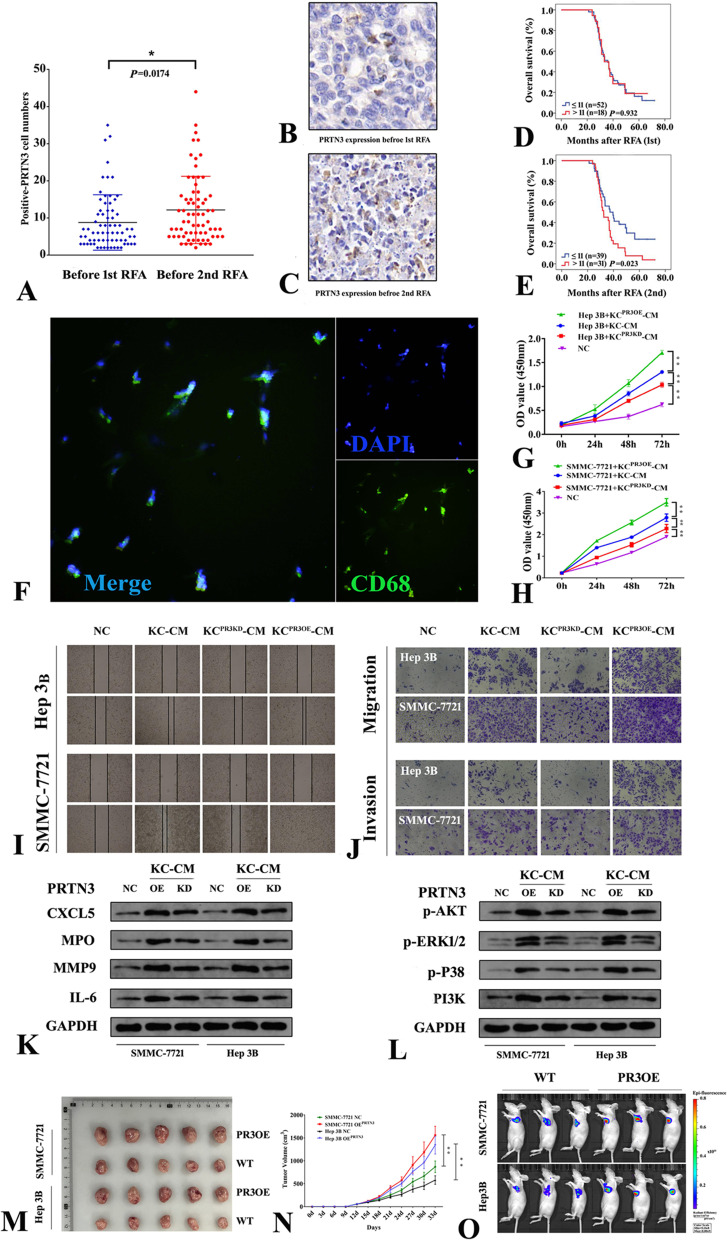
The role of predictive survival and tumorigenesis effects of PRTN3 in heat stress treated HCC. **A** Immunohistochemical analysis indicated that the expression of PRTN3 in HCC was increased after RFA. **B**-**C** Representative immunohistochemical images of PRTN3 expression in HCC tissues before the first RFA treatment (**B**) and before the second RFA treatment (**C**). **D**-**E** Kaplan‒Meier analysis showing the OS predictive abilities of PRTN3 before RFA treatment (**D**) and after RFA treatment (**E**) in patients with early recurrence of HCC. **F** Immunofluorescence staining of DAPI and CD68 in KCs. **G**-**H** Cell proliferation assay of Hep 3B and SMMC-7721 after heat stress treatments and cultured with KC-CM transfected by lentiviral vector for PRTN3-overexpression (PR3OE) or PRTN3-knockdown (PR3KD). **I** Wound-healing assay. **J** Cell migration and invasion assays by transwell. **K** Western blot analysis of protein expression levels of CXCL5, MPO, MMP9 and IL-6 in heat stress HCC cells as were affected by KC-CM after PR3OE or PR3KD. **L** Western blot analysis of protein expression of p-AKT, p-ERK1/2, p-P38 and PI3K in heat stress HCC cells affected by KC-CM after PR3OE or PR3KD. **M** Picture of xenograft tumors were shown in the PR3OE group and wild type group (*n* = 5). N: The growth curves of each group of xenograft tumors were displayed. **O** The xenograft mice models in vivo were analyzed by IVIS to identify the potential of PRTN3 in promoting tumor grow. All data are expressed as the mean ± SD of three independent experiments. **p < 0.01

To investigate the association between PRTN3 and cancer progression promoted by RFA in patients with HCC, we analyzed the predictive power of PRTN3 in the OS of early recurrent HCC following RFA. The baseline characteristics of 70 patients with recurrence after RFA are shown in Table 2. Immunohistochemical analysis revealed higher expression of PRTN3 in cancer tissues of HCC with early recurrence after RFA than in tissues from before RFA (*p* = 0.0174, Fig. 6A-C). The expression levels of PRTN3 before the 1st RFA treatment could not predict the OS of HCC patients (*p* = 0.932, Fig. 6D). However, after RFA treatment, high PRTN3 expression was associated with unfavorable OS (*p* = 0.023, Fig. 6E) in patients with early recurrence of HCC following RFA, probably suggesting an important protumor effect of PRTN3 induced by RFA.

**Table 2:** Baseline characteristics of 70 recurrent patients after RFA

Characteristics	RFA cohort
Age, yr, median, (range)	51.0 (31–79)
Gender (Female/Male)	13/57 (18.6%/81.4%)
Cirrhosis (yes/no)	57/13(81.4%/18.6%)
GGT, U/L, median (range)	53.5 (17.0–500.0)
ALT, U/L, median (range)	42.5 (9.0–499.0)
ALB, g/L, median (range)	45.0 (33.0–57.0)
TBIL, µmol/L, median (range)	12.6 (4.6–31.6)
AFP, ng/ml, median (range)	100.5 (1–60,500.0)
HBsAg (Positive/Negative)	59/11 (84.3%/15.7%)
Tumor number (single/multiple)	59/11 (84.3%/15.7%)
Tumor size (cm)	
≤ 2	10 (14.3%)
> 2, ≤ 3	23 (32.9%)
> 3, ≤ 5	37 (52.8%)
BCLC stage (0/A)	7/63 (10.0%/90.0%)

*Abbreviations*: *RFA* Radiofrequency ablation, *GGT* Gamma-glutamyl transpeptidase, *ALB* Albumin, *TBIL* Total bilirubin, *AFP* Alpha fetoprotein, *HBsAg* Hepatitis B virus surface antigen, *BCLC* Barcelona Clinic Liver Cancer

### PRTN3 expressed in KCs promotes sublethal heat stress-treated HCC cell proliferation, migration and invasion

The single-cell sequencing of PRTN3^+^ cells in liver tissues from The Human Protein Atlas database (https://www.proteinatlas.org/ENSG00000196415-PRTN3/single+cell+type/liver) confirmed that sources of PRTN3 were mainly T cells, KCs and hepatocytes (Figure S8). To further validate the effects of PRTN3 in the HCC milieu and the interaction between KCs and tumor cells induced by PRTN3, we isolated human KCs from normal liver tissues (Fig. 6F). After coculture with KC-CM for 24 h, the proliferative abilities of the two sublethal heat stress-treated HCC cell lines were increased compared with those of the normal control cells cultured with DMEM (*P* < 0.01, Fig. 6G-H). Compared with the KC-CM group, knockdown of PRTN3 expression in KCs (KC^PR3KD^-CM) attenuated heat stress-induced HCC cell proliferation, whereas overexpression of PRTN3 in KCs (KC^PR3OE^-CM) promoted heat stress-induced HCC cell proliferation (*p* < 0.01, Figure S9). Similarly, wound healing (Fig. 6I and Figure S10A) and transwell assays (Fig. 6J and Figure S10B-C) showed that KC^PR3KD^-CM could alleviate heat stress-induced migration and invasion of HCC cells, respectively. Conversely, KC^PR3OE^-CM could promote the migration and invasion of heat stress-treated HCC cells. These data may reveal that PRTN3 expressed in KCs plays a critical role in HCC progression.

### PRTN3 promotes sublethal heat stress-induced tumor growth via multiple oncogenic factors and the PI3K/AKT and P38/ERK signaling pathways

To investigate the molecular mechanisms by which PRTN3 expressed in KCs affects heat stress (in vitro simulated RFA)-treated HCC, the protein expression levels of several oncogenic factors in the two HCC cell lines were detected. Compared with KC-CM, KC^PR3OE^-CM promoted the protein expression levels of CXCL5, MPO, MMP9 and IL-6 in HCC cells. However, compared to KC^PR3OE^-CM, KC^PR3KD^-CM reduced their expression levels (*p* < 0.01, Fig. 6K and Figure S10D-G). Moreover, KC^PR3OE^-CM induced the expression and phosphorylation of PI3K/AKT and P38/ERK signaling factors (*p* < 0.01, Fig. 6L and Figure S10H-L). After we established a xenograft mouse model, analysis of the two sublethal heat stress-treated HCC cell-induced tumors showed that tumors injected with lentiviral vectors for PR3OE had a higher tumor burden and grew faster than those injected with wild-type vectors (Fig. 6M-O). Collectively, these observations indicate that overexpression of PRTN3 exerts an oncogenic role in HCC cells via multiple oncogenic factors and the PI3K/AKT and P38/ERK signaling pathways, partially through interaction with Kupffer cells.

## Discussion

Increasing evidence has shown that iRFA can induce an immune response and tumor progression and recurrence at the periablational “red zone” [11, 13, 14, 20–22, 35]. Due to the unavailability of human HCC tissue samples after RFA, the relevant viewpoints established with research on animal models and human peripheral blood remain to be supported by deeper rationale and reliable evidence. To the best of our knowledge, the current study is the first attempt to comprehensively dissect the immune response- and tumor relapse-related mechanisms in residual HCC after iRFA by assessing quantitative changes in immune cells and performing integrated transcriptomic and proteomic analysis. First, we found that the localized disruption induced by RFA led to the recruitment of a variety of tumor-promoting circulating immune cells and led to an imbalance between antitumor immune cells, promoting tumor development. Second, transcriptomic and proteomic analyses revealed distinct biological processes, metabolic processes and oncogene signaling pathway activation at different molecular levels after iRFA. Interestingly, there were more translational regulation-related genes dysregulated at the mRNA level than at the protein level. One possible explanation is that increased transcription of genes acts as a compensatory mechanism for protein degradation [36]. Third, we identified PRTN3 as a frequently mutated driver gene in iRFA-triggered HCC progression and recurrence.

Heat-ablated lesions are assumed to have three zones: a central zone with temperature > 60 °C, a transitional zone with temperature approximately 43–50 °C, and the ablation unaffected surrounding tissue [37]. After iRFA, the tumor cells in the transitional zone could undergo sublethal injury and lead to rapid tumor progression. Our results suggest that infiltrating lymphocytes (TILs) in the transitional zone may promote the spread of cancer cells. However, the number of TILs did not dramatically increase immediately after iRFA, likely because of the “delayed effect” that occurred as a result of mutual reactions between the TILs and local microenvironment factors, such as angiogenesis-related factors and extracellular matrix components [38]. In some patients, we still observed increased recruitment of local CD8^+^ T cells, CD19^+^ cells, and CD56^+^ cells in the transitional zone, probably resulting in extensive intratumor heterogeneity, which could facilitate the development of multiple tumor subclones under the ongoing effects of TILs. The systemic immune response exhibited a diversity of quantitative changes. The present study provided evidence showing that the balance of antitumor lymphocytes was disrupted, as reflected by the increase in CD8^+^ T cells and Tregs and the decrease in CD16^+^CD56^+^ NK cells; these changes could facilitate the dissemination of cancer cells if tumor immune surveillance and defense were substantially attenuated. On the other hand, the combination of immunotherapy with the RFA approach, which could manipulate the local immune status in the TME by modulating multiple functions of TILs, may be an appropriate strategy.

Both transcriptomic and proteomic analyses revealed distinct variations in immune status and tumor invasion-associated molecular function and signaling pathways at the gene and protein levels. In periablational lesions, the proinflammatory cytokines released from the ablated tumoral and peritumoral tissues can enhance the secretion of factors by various cell types, thereby leading to a persistent inflammatory cascade [25]. Moreover, prevalent variation events, such as AS, INDEL, SNP, and fusion events in DEGs, and subcellular localization changes in DEPs occurred in response to HCC microenvironment changes induced by iRFA stimulation. Furthermore, our integrated analysis of HCC biopsies revealed distinct changes in biological processes, metabolic reprogramming events and signaling pathway activation at different molecular levels. After RFA, we observed enrichment of tumor growth- and immune/inflammatory response-associated GO terms and signaling pathways (such as several binding processes, positive regulation of NF-kappaB transcription factor activity, neutrophil chemotaxis, positive regulation of the inflammatory response, and the IL-17 signaling pathway) and dysregulation of protein expression, suggesting that the different statuses of DEGs and DEPs during posttranslational processes and the malignant functional transformation of DEGs were significantly associated with local tumor inflammation/immunity. These results provide a novel understanding of immunometabolism induced by iRFA and possible targets to regulate the antitumor response. In the protein dimension, this integrated analysis also reflected cell behavior alterations due to posttranscriptional and posttranslational regulation and the feedback regulation of cell signaling pathways.

PRTN3 is one of the primary components of neutrophil azurophilic granules [39] and a leukemia-associated antigen specifically recognized by CD8^+^ cytotoxic T-lymphocytes (CTLs) [40]. In some clinical situations (e.g., leukemia and Wegener’s disease), PRTN3_235_, one peptide epitope, is recognized by CD4^+^ T cells and Tregs in immune responses against PRTN3 as a naturally processed HLA-class-II-epitope; this peptide is also recognized by dendritic cells in the blood of healthy individuals [40]. These results were consistent with our bioinformatics analysis results showing that PRTN3 expression was positively correlated with the infiltration of CD8^+^ T cells, CD4^+^ T cells and dendritic cells. The PRTN3-related immune response could participate in tumor progression, which was supported by several studies. For instance, the inflammatory response triggered by vulvovaginal microflora may employ PRTN3 in association with the progression of squamous cell vulvar carcinoma (VSCC), which has an aggressive phenotype and may be used for the stratification of patients with this disease [41]. A potential immunotherapy approach for acute myeloid leukemia (AML) may be implemented through the application of HLA ligands, which could allow several established AML-related antigens, including PRTN3, to attract additional CD4^+^ T-cell epitopes [42]. Similar to our results, high PRTN3 expression also predicted poor prognosis in clear cell renal cell carcinoma [43] and pancreatic cancer [44]. The data from our study (Fig. 2) and the single-cell analysis of PRTN3^+^ cells in liver tissues (Figure S8) showed that the immune response (e.g., T cells, B cells and DCs) was induced by RFA and that the main sources of PRTN3 were T cells, KCs and hepatocytes. As important antigen-presenting cells, KCs may have direct (via tumor cells) or indirect (via T cells) promoting tumor effects. Here, we first explored the direct tumor-promoting roles of PRTN3 expressed in KCs and hepatocytes. Our functional experiments also demonstrated that PRTN3 may drive cancer migration and metastasis via interactions between heat stress-treated HCC cells and KCs, which are liver-resident immune cells. After overexpression of PRTN3 in HCC cells under heat stress, multiple oncogenic factors and signaling pathways are activated and contribute to tumor growth. Consequently, PRTN3 may be a hub gene involved in the immune response-associated molecular networks occurring in the TME, at least in the inflamed HCC microenvironment after iRFA. In the future, the crosstalk between T cells, especially CD8^+^ T cells, and tumor cells needs to be investigated in depth.

We acknowledge the limitations of the current study. (i) The sample size of the omics studies and prognostic analyses is small due to our strict inclusion criteria in this novel clinical study design and the unavailability of HCC tissue samples after RFA. (ii) A further elaborate investigation of signaling pathways such as the IL-17 and MPO-PTRN3 pathways should be performed by combining basic and clinical research according to the action characteristics of RFA. (iii) With enough iRFA-affected HCC tissue specimens, other multiomics studies, such as metabolomics, phosphoproteomics, whole-exome sequencing, and single-cell sequencing, could provide a more comprehensive report to evaluate tumor heterogeneity, distinct molecular subtypes and immunophenotypes with different clinical and molecular features.

## Conclusions

In summary, we confirmed that complex translationally regulated genes and protein networks intertwining with inflammatory/immune responses induced by iRFA drive the dissemination and invasion of cancer cells. Based on the findings, we believe that iRFA-induced local immunity might not only be a seedbed of tumor recurrence but might also be a suitable target for targeted drugs, which could be combined with immunotherapy for the treatment of HCC. Further studies using large cohorts with comprehensive multiomics explorations are warranted to comprehensively clarify the mechanisms of tumor progression and establish a close connection between the immune responses and the treatment strategy of HCC after RFA.

## Supplementary Information

**Additional file 1 Figure S1.** Sequence data qualitycontrol before transcriptomics analysis. A-B: Representativeimagines showing error rate distribution along sequencedata (reads). C-D: Representative pie graphs showing the classification of raw readsfilter. E-F: Representative imagines showing bases content alongsequence reads. **Figure S2.** Genomemapping, gene expression distribution and pearson correlation between samples.A-B: Representative pie graphs showing thepercentage of genome regions based on the reads mapping. C: box plotdisplaying the distribution of gene expression levels in different samplesafter calculating the expression value (FPKM) of all genes in each sample. D:Pearson correlation between samples according to FPKMvalue. **Figure S3.** Thealternative splicing events. Analysis of rMATS software showingdifferential alternative 3′ splice site (A3SS) event of UBXN11 (A) and skippedexon (SE) events of AHI1 (B), ENTR1 (C), IQCB1 (D), KIAA1191 (E), LCN12 (F),LRR1 (G) and ZNF26 (H) in different samples. RFA test representsthe group with iRFA treated patient; RFA Ctrl represents the group without iRFAtreated patient. **Figure S4.** The frequency ofinsertion-deletion (INDEL) and single nucleotide polymorphisms (SNP)alterations in various genes. A-B: Impact andregion of INDEL. C-E: Impact (C)function (D) and region (E) of SNP. **Figure S5.** Circos plots of fusion gene events in eachsample. **Figure S6.** Protein qualitative and sample pepeatabilitydetection. A: Histogram 0f protein identification results showing2014926 total spectrums, 879371 matched spectrums, 76509 peptides, 72533 uniquepeptides and 7380 protein groups were identified. B: Boxplot showing therelative standard deviation of protein quantification values between two groupsof samples. C: Pearson’s Correlation Coefficient between two samples. **Figure S7.** PPI network analysis ofPRTN3 and the association with immune cells. A: PPI network analysis of PRTN3 based on the STRINGdatabase. B-C: PPI network analyses of PRTN3 in HCC (B) and adjacent tumortissues (C). D: The relationship between PRTN3 and immune infiltration in HCCusing the TIMER online tool based on TCGA data. E: The links between genomicaberrations of PRTN3 and the abundance of TIICs by the "SCAN" modulein the TIMER database. **Figure S8.** The single-cell sequencing of PRTN3^+^ cells in livertissues from The Human Protein Atlas database. **Figure S9.** Expression of PRTN3detected by qRT-PCR in HCC cell lines (A, Hep 3B and SMMC-7721) and Kupffercells (B). **Figure S10.** Migrative and invasive abilities analysis and the relative proteinexpression levels of western blotting. A:Migrative abilities analysis of HCC cell after cultured with KC-CM transfected for PR3OE or PR3KD. B-C: Invasive and migrative abilities of HCCcell after cultured with KC-CM transfected for PR3OE or PR3KD according to transwell assay analysis. D-L: The relative protein expression levels of CXCL5(D), MPO (E), MMP9 (F), IL-6 (G), p-AKT (H), p-ERK1 (I), p-ERK2 (J), p-P38 (K)and PI3K (L), respectively.All data are presented as the means ± SD of three independent experiments. *p< 0.01. **Table S1.** 389 dysregulated genes detected detected in two groups. **Table S2.** 20 dysregulated proteinsdetected in two groups. **Table S3.**Proteins with significantly differential expression in subcellular location. **Table S4.** Transcription factorassociated proteins. **Table S5.**Integrated data of transcriptome and proteome for GO and KEGG pathwayenrichment analysis.

## Data Availability

All data relevant to this study are available from the corresponding authors upon reasonable request. RNA-seq data can be accessed on NIH GEO (GSE212604, https://www.ncbi.nlm.nih.gov/geo/). The mass spectrometry proteomics data have been deposited to the ProteomeXchange Consortium (http://proteomecentral.proteomexchange.org) via the iProX partner repository with the dataset identifier PXD036767.

## Abbreviations

HCC: Hepatocellular carcinoma
iRFA: Incomplete radiofrequency ablation
cRFA: Complete RFA
DEGs: Differentially expressed genes
DEPs: Differentially expressed proteins
PRTN3: Proteinase-3
OS: Overall survival
cRFA: Complete RFA
LRT: Locoregional therapy
VEGF: Vascular endothelial growth factor
HGF: Hepatocyte growth factor
TACE: Transarterial chemoembolization
HAIC: Hepatic artery infusion chemotherapy
DCs: Dendritic cells
CTLA-4: T-lymphocyte-associated protein 4
TME: Tumor microenvironment
BCLC: Barcelona Clinic Liver Cancer
PBMC: Peripheral blood mononuclear cells
GO: Gene Ontology
KEGG: Kyoto Encyclopedia of Genes and Genomes
WGCNA: Weighted correlation network analysis
PPI: Protein–Protein Interactions
FDR: False discovery rate
HCCDB: Hepatocellular Carcinoma Database
STRING: Search Tool for the Retrieval of Interacting Genes
TIMER: Tumor Immune Estimation Resource
SCNA: Somatic copy number alteration
TMA: Tissue microarray
IHC: Immunohistochemistry
BP: Biological process
CC: Cellular component
MF: Molecular function
TIL: Tumor infiltrating lymphocyte
CTLs: Cytotoxic T-lymphocytes
KCs: Kupffer cells
qRT-PCR: Quantitative real-time polymerase chain reaction
CM: Conditioned medium
