# Supplementary material for: Integrated proteogenomic characterization reveals an imbalanced hepatocellular carcinoma microenvironment after incomplete radiofrequency ablation
Source: J Exp Clin Cancer Res. 2023 May 25;42:133. doi: 10.1186/s13046-023-02716-y (PMC10210354; doi:10.1186/s13046-023-02716-y)
Supplement: Supplementary file 1 — Additional file 1 Figure S1. Sequence data qualitycontrol before transcriptomics analysis. A-B: Representativeimagines showing error rate distribution along sequencedata (reads). C-D: Representative pie graphs showing the classification of raw readsfilter. E-F: Representative imagines showing bases content alongsequence reads. Figure S2. Genomemapping, gene expression distribution and pearson correlation between samples.A-B: Representative pie graphs showing thepercentage of genome regions based on the reads mapping. C: box plotdisplaying the distribution of gene expression levels in different samplesafter calculating the expression value (FPKM) of all genes in each sample. D:Pearson correlation between samples according to FPKMvalue. Figure S3. Thealternative splicing events. Analysis of rMATS software showingdifferential alternative 3′ splice site (A3SS) event of UBXN11 (A) and skippedexon (SE) events of AHI1 (B), ENTR1 (C), IQCB1 (D), KIAA1191 (E), LCN12 (F),LRR1 (G) and ZNF26 (H) in different samples. RFA test representsthe group with iRFA treated patient; RFA Ctrl represents the group without iRFAtreated patient. Figure S4. The frequency ofinsertion-deletion (INDEL) and single nucleotide polymorphisms (SNP)alterations in various genes. A-B: Impact andregion of INDEL. C-E: Impact (C)function (D) and region (E) of SNP. Figure S5. Circos plots of fusion gene events in eachsample. Figure S6. Protein qualitative and sample pepeatabilitydetection. A: Histogram 0f protein identification results showing2014926 total spectrums, 879371 matched spectrums, 76509 peptides, 72533 uniquepeptides and 7380 protein groups were identified. B: Boxplot showing therelative standard deviation of protein quantification values between two groupsof samples. C: Pearson’s Correlation Coefficient between two samples. Figure S7. PPI network analysis ofPRTN3 and the association with immune cells. A: PPI network analysis of PRTN3 based on the STRINGdatabase. B-C: PPI network analyses of PRTN3 [file 13046_2023_2716_MOESM1_ESM.docx]

**Supplementary information**

**Supplementary Figures**


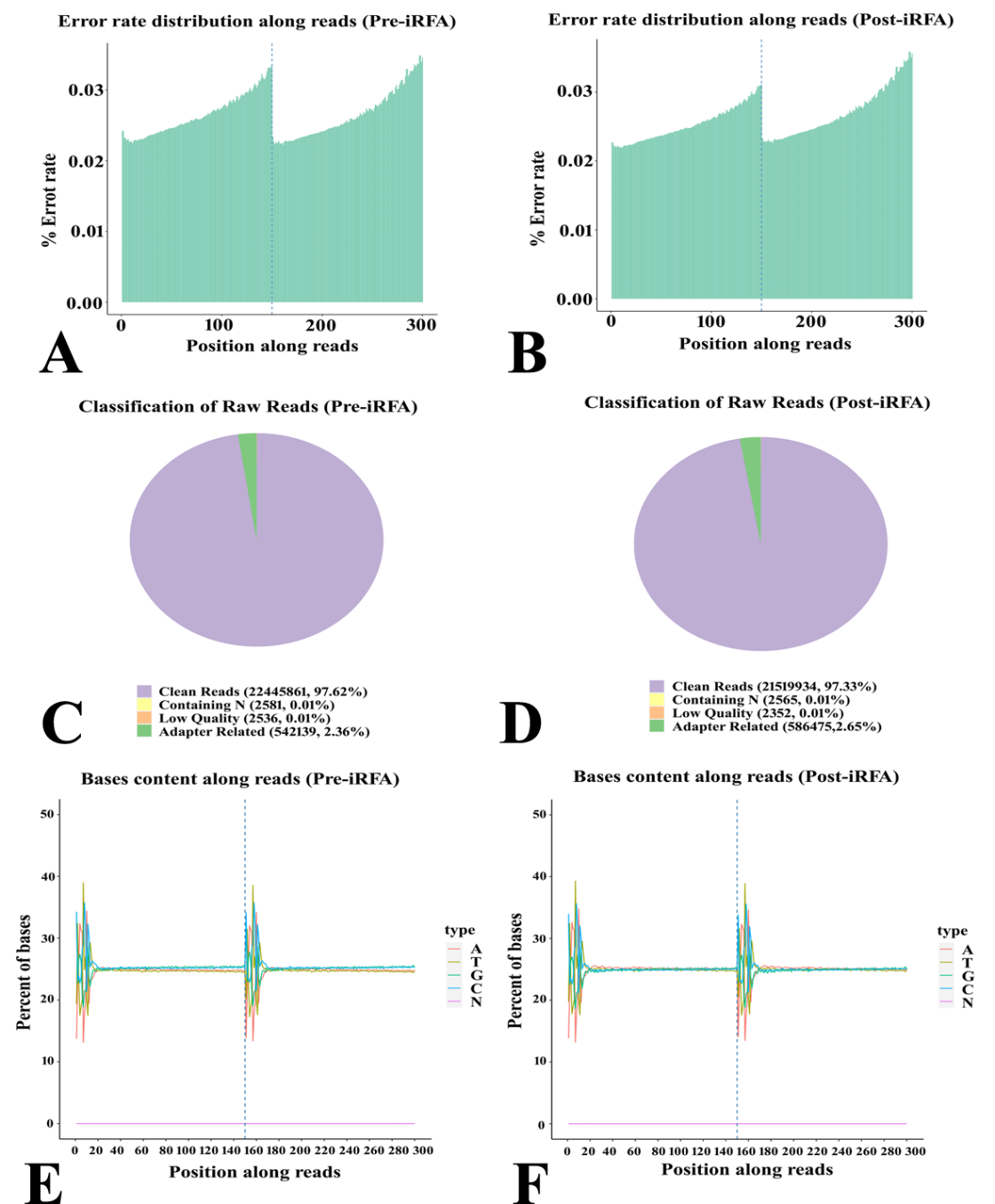


**Figure S1 Sequence data quality control before transcriptomics analysis.** A-B: Representative imagines showing error rate distribution along sequence data (reads). C-D: Representative pie graphs showing the classification of raw reads filter. E-F: Representative imagines showing bases content along sequence reads.


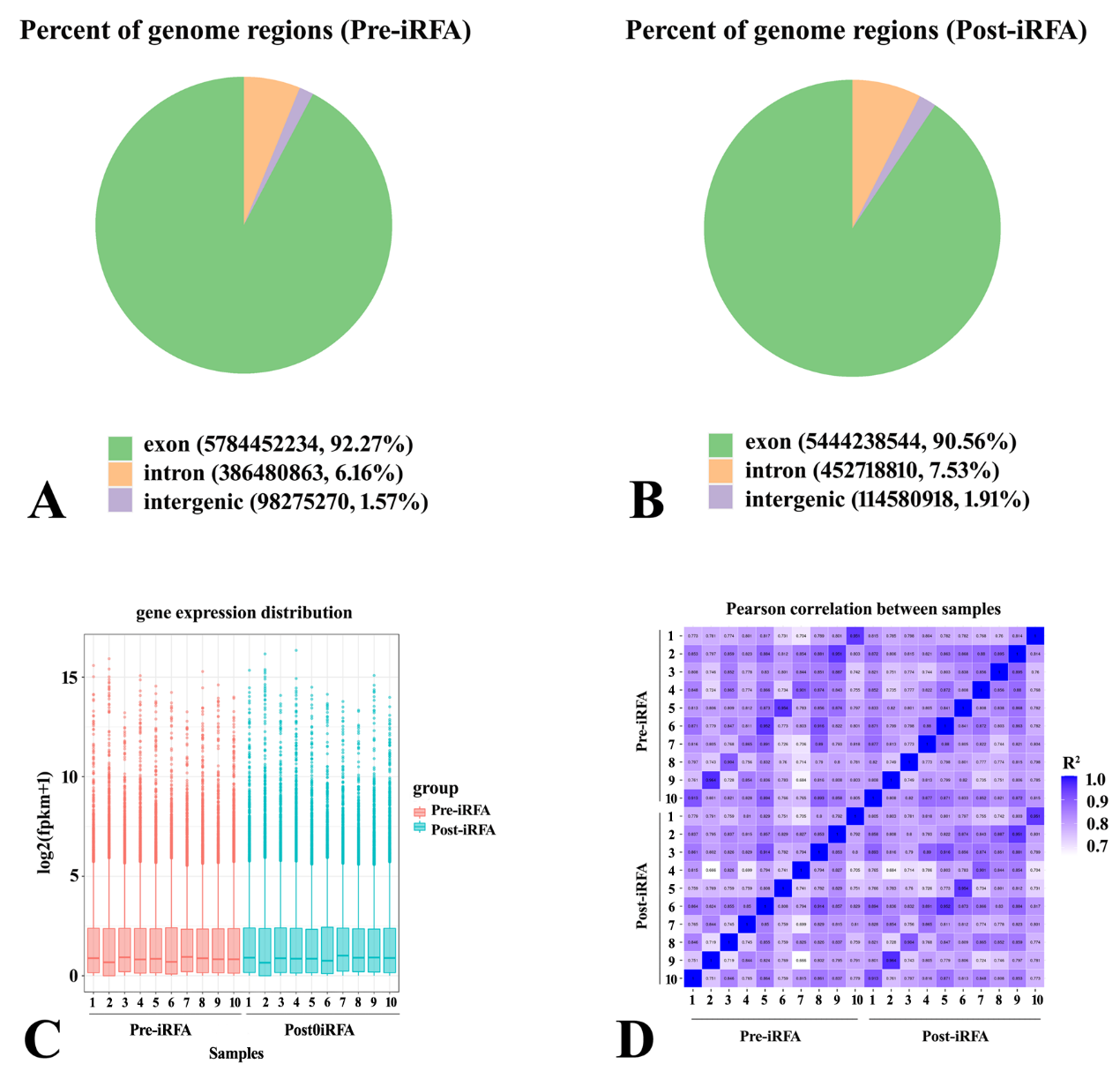


**Figure S2 Genome mapping, gene expression distribution and pearson correlation between samples.** A-B: Representative pie graphs showing the percentage of genome regions based on the reads mapping. C: box plot displaying the distribution of gene expression levels in different samples after calculating the expression value (FPKM) of all genes in each sample. D: Pearson correlation between samples according to FPKM value.


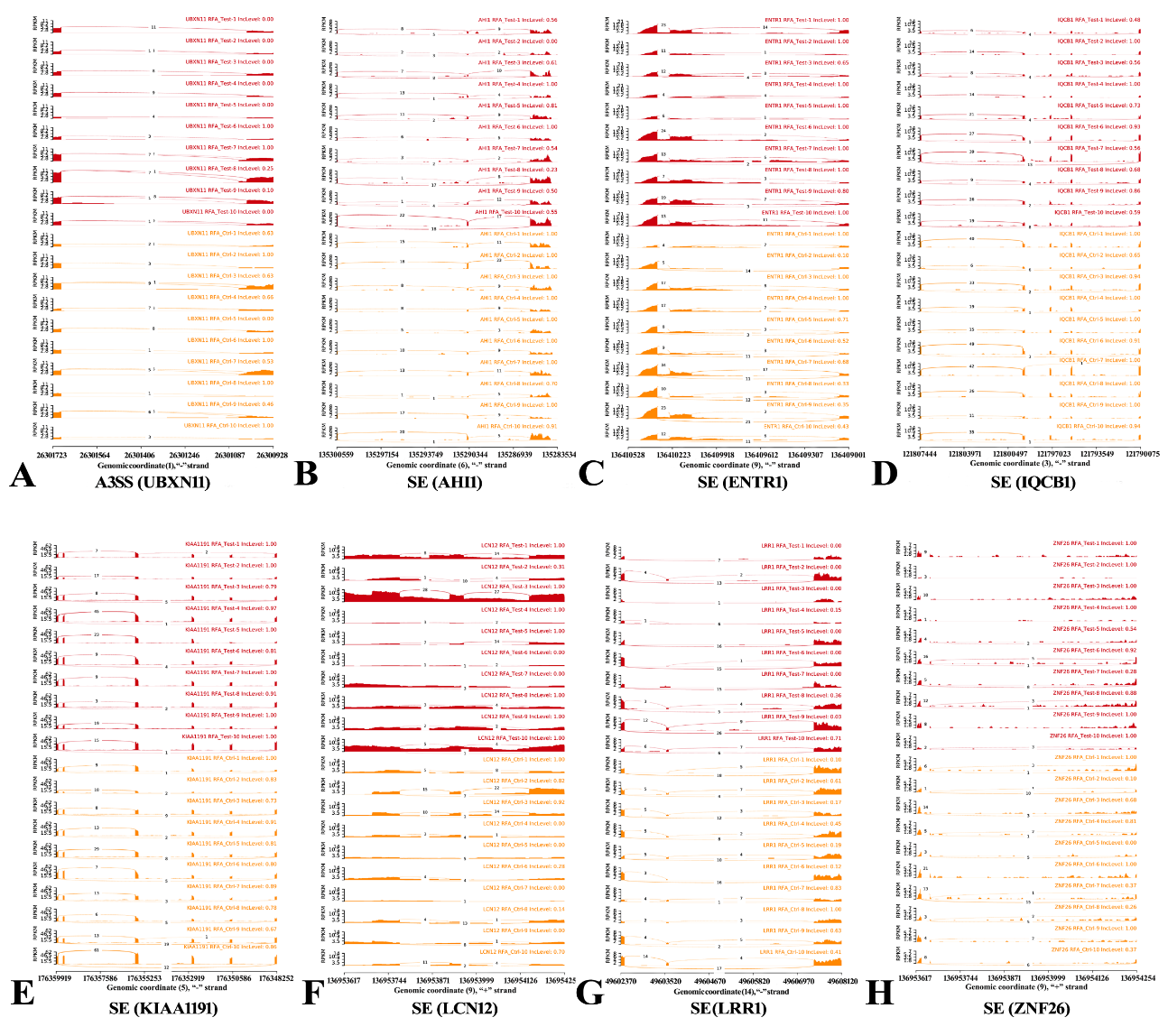


**Figure S3 The alternative splicing events.** Analysis of rMATS software showing differential alternative 3′ splice site (A3SS) event of UBXN11 (A) and skipped exon (SE) events of AHI1 (B), ENTR1 (C), IQCB1 (D), KIAA1191 (E), LCN12 (F), LRR1 (G) and ZNF26 (H) in different samples. RFA test represents the group with iRFA treated patient; RFA Ctrl represents the group without iRFA treated patient.


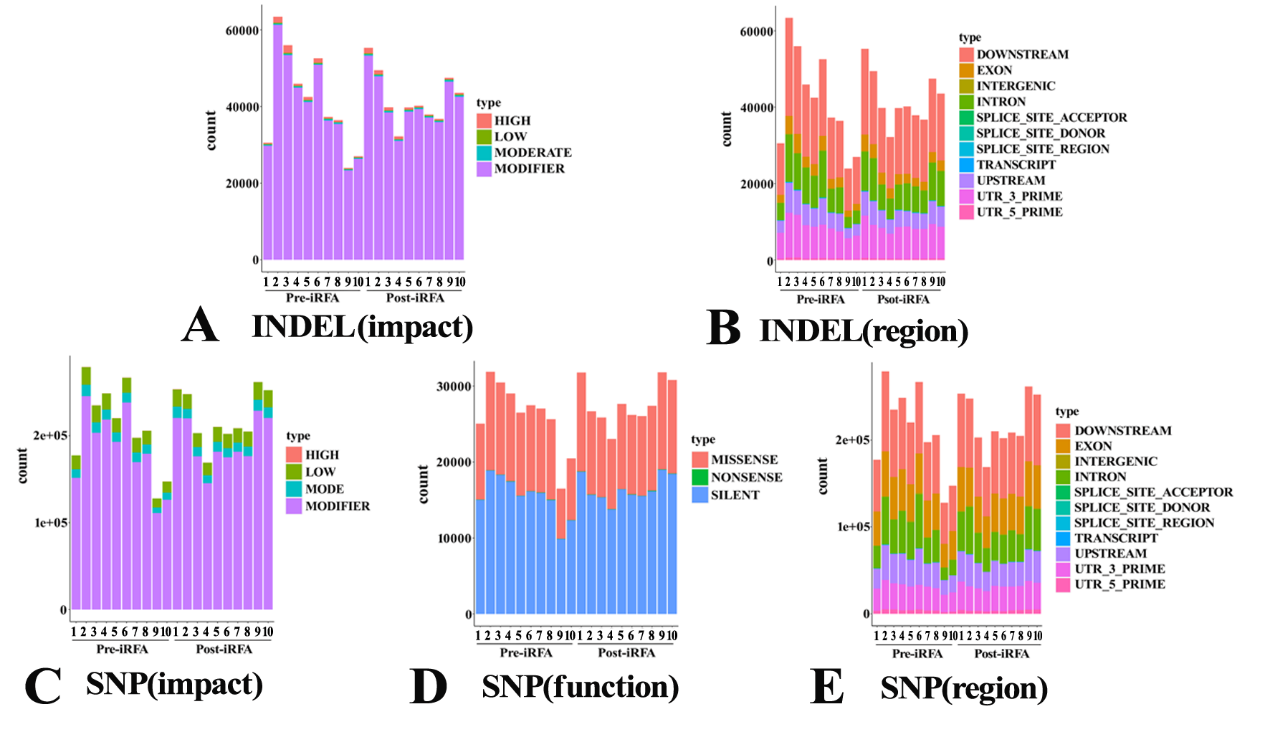


**Figure S4 The frequency of insertion-deletion (INDEL) and single nucleotide polymorphisms (SNP) alterations in various genes.** A-B: Impact and region of INDEL. C-E: Impact (C)function (D) and region (E) of SNP.


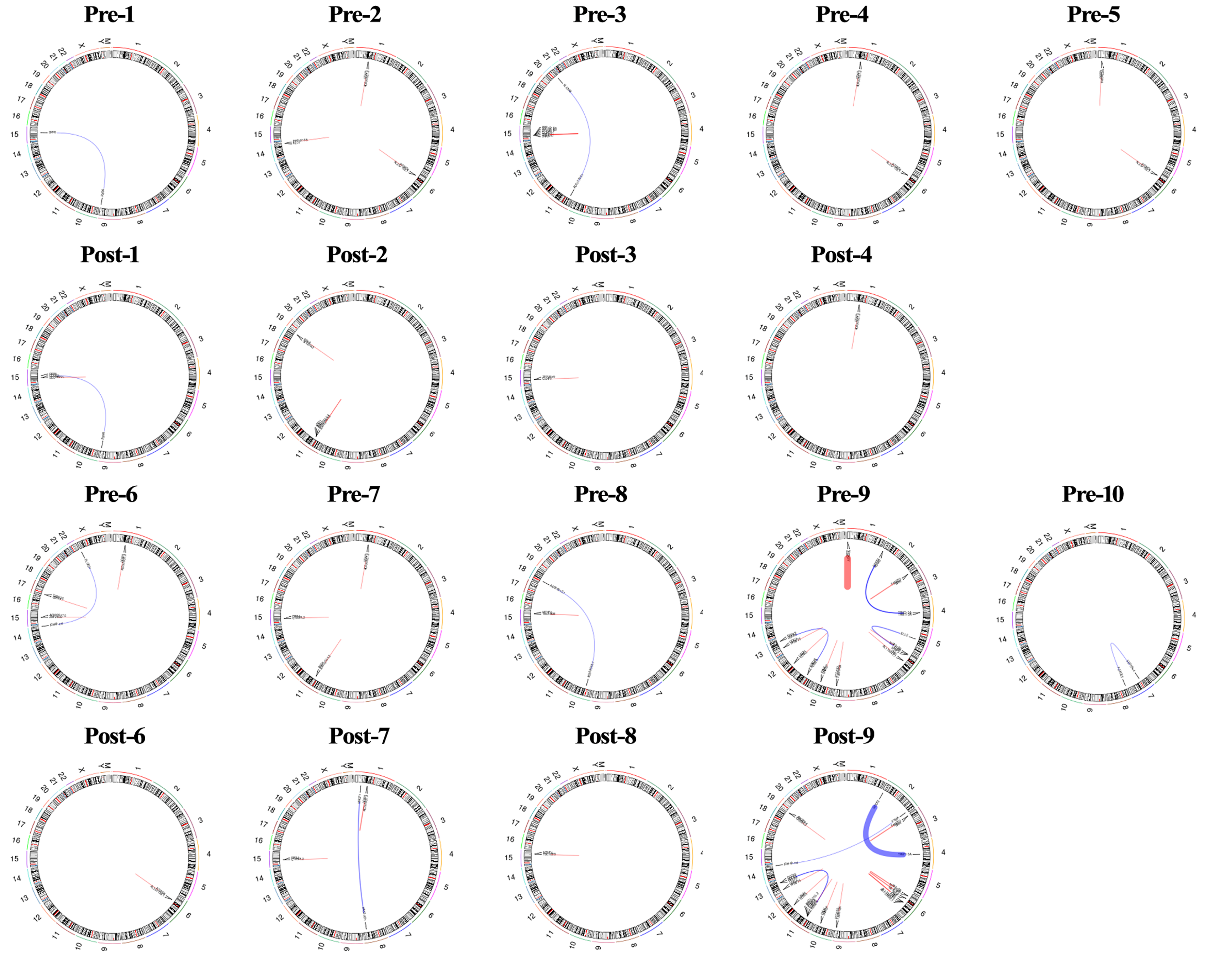


**Figure S5 Circos plots of fusion gene events in each sample.**


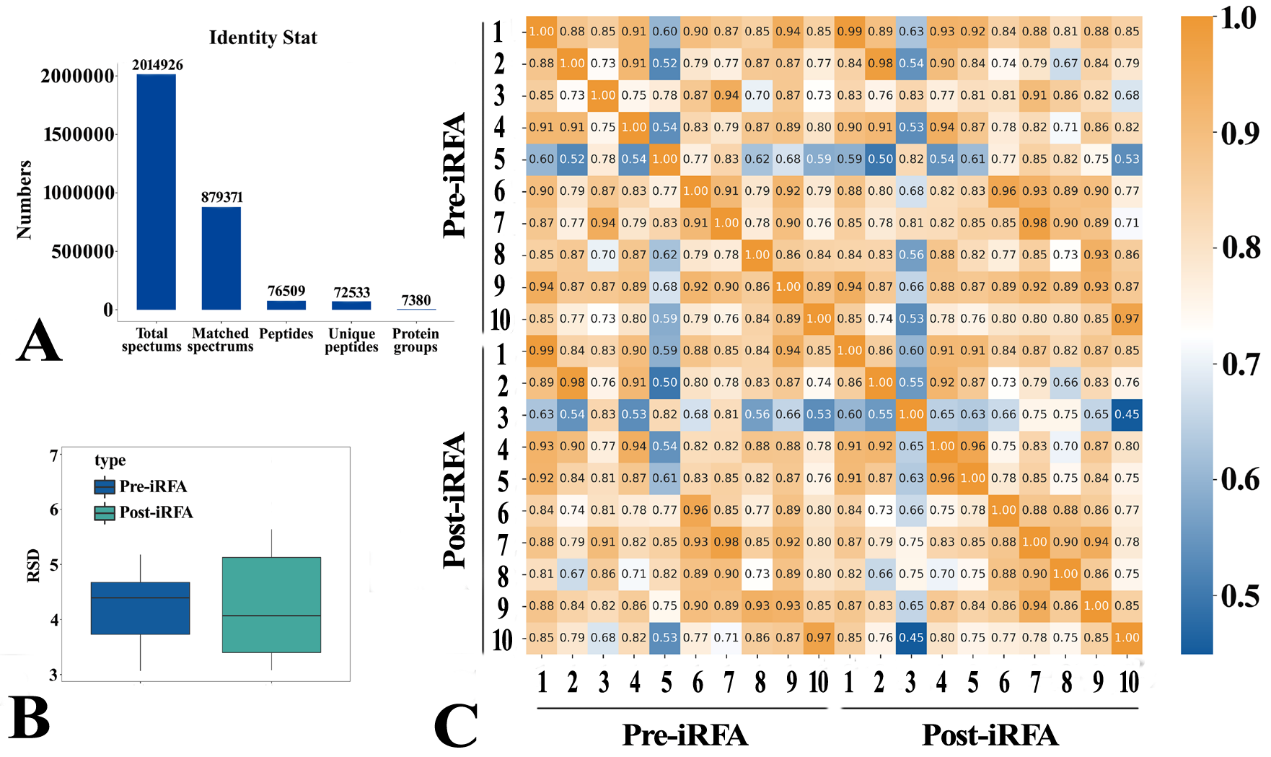


**Figure S6 Protein qualitative and sample pepeatability detection.** A: Histogram 0f protein identification results showing 2014926 total spectrums, 879371 matched spectrums, 76509 peptides, 72533 unique peptides and 7380 protein groups were identified. B: Boxplot showing the relative standard deviation of protein quantification values between two groups of samples. C: Pearson’s Correlation Coefficient between two samples.


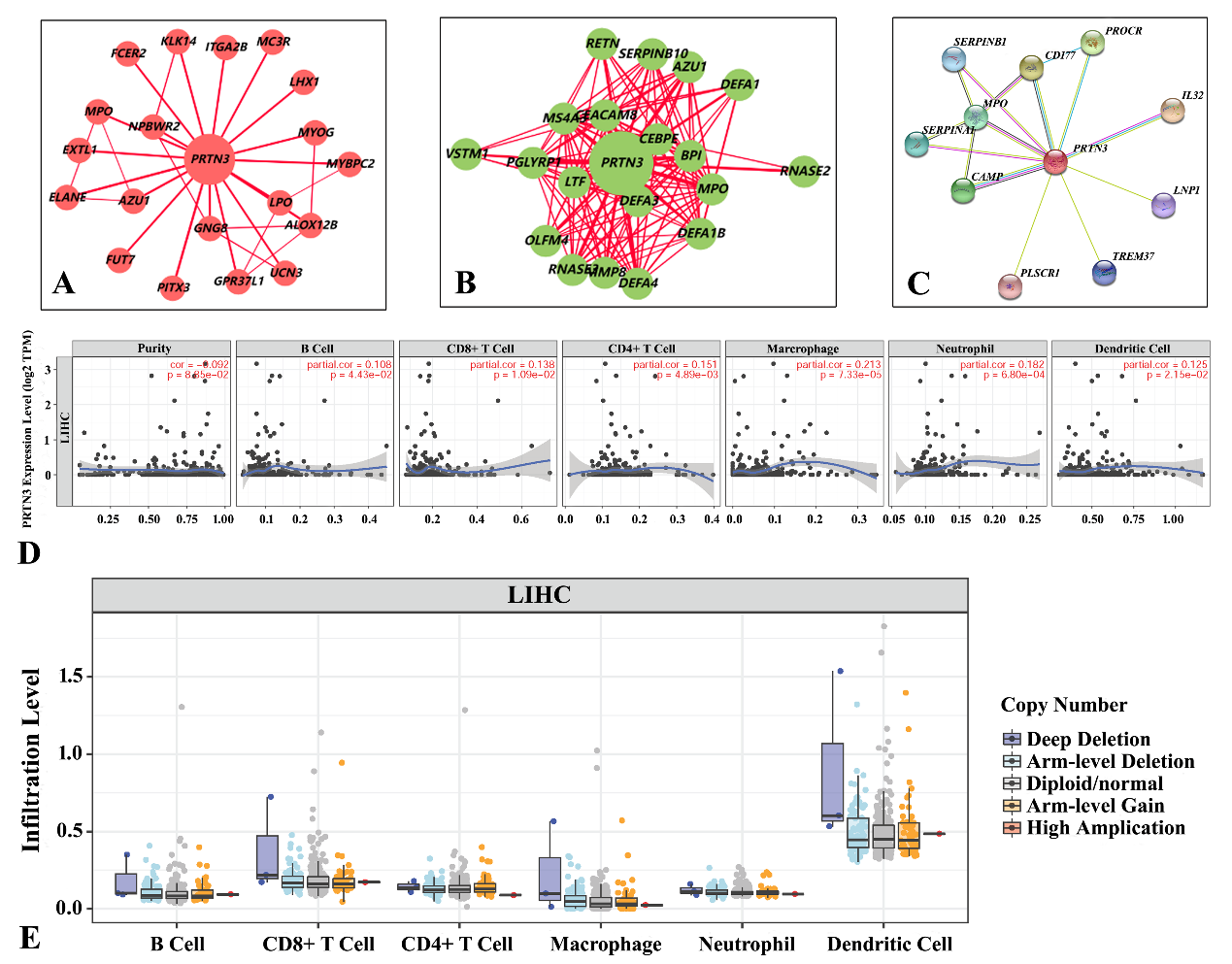


**Figure S7 PPI network analysis of PRTN3 and the association with immune cells.** A: PPI network analysis of PRTN3 based on the STRING database. B-C: PPI network analyses of PRTN3 in HCC (B) and adjacent tumor tissues (C). D: The relationship between PRTN3 and immune infiltration in HCC using the TIMER online tool based on TCGA data. E: The links between genomic aberrations of PRTN3 and the abundance of TIICs by the "SCAN" module in the TIMER database.


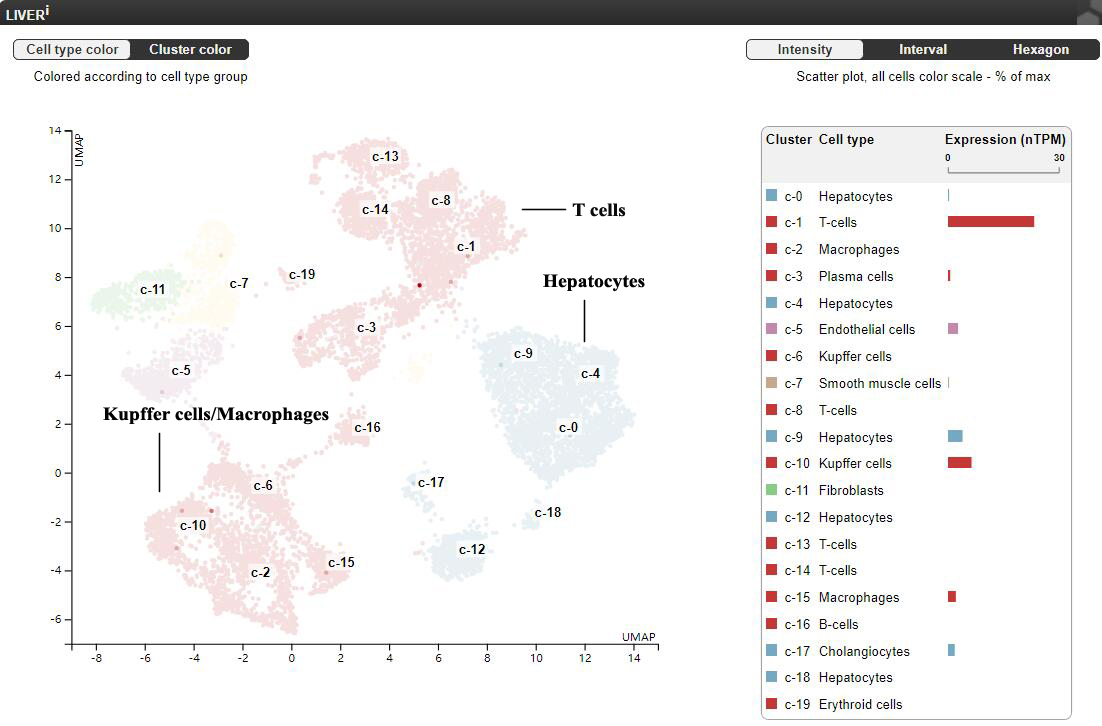


**Figure S8** The single-cell sequencing of PRTN3^+^ cells in liver tissues from The Human Protein Atlas database.


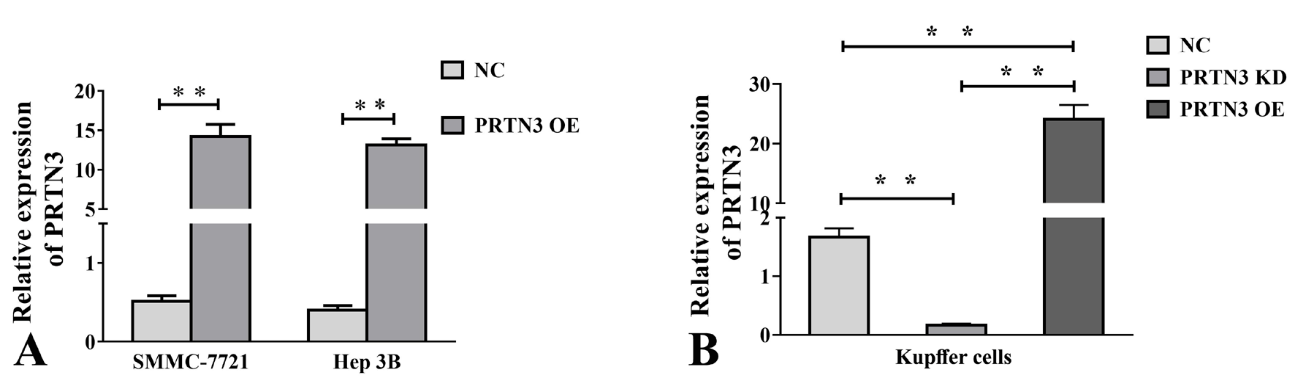


**Figure S9 Expression of PRTN3 detected by qRT-PCR in HCC cell lines (A, Hep 3B and SMMC-7721) and Kupffer cells (B)**


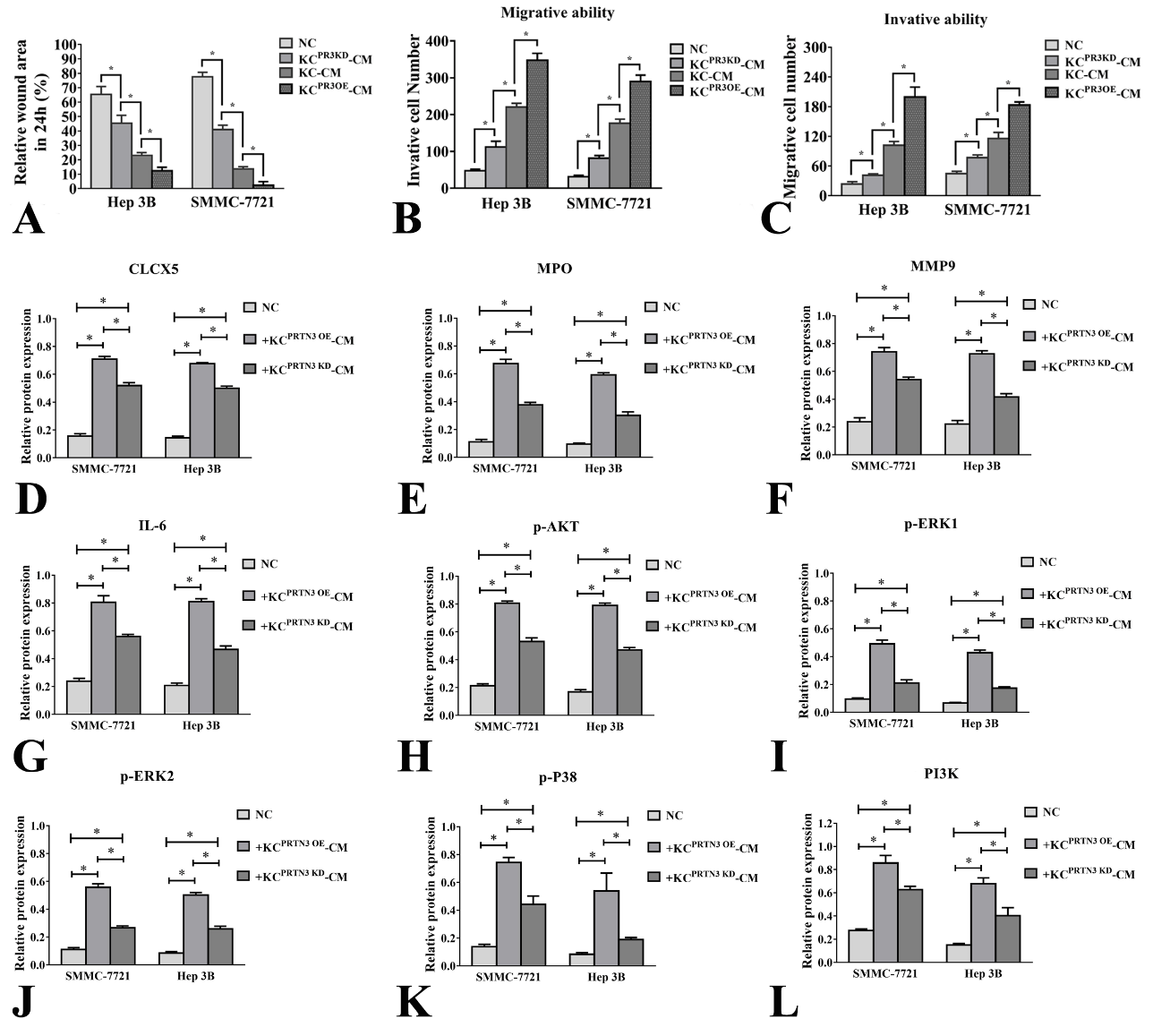


**Figure S10 Migrative and invasive abilities analysis and the relative protein expression levels of western blotting.** A: Migrative abilities analysis of HCC cell after cultured with KC-CM transfected for PR3OE or PR3KD. B-C: Invasive and migrative abilities of HCC cell after cultured with KC-CM transfected for PR3OE or PR3KD according to transwell assay analysis. D-L: The relative protein expression levels of CXCL5 (D), MPO (E), MMP9 (F), IL-6 (G), p-AKT (H), p-ERK1 (I), p-ERK2 (J), p-P38 (K) and PI3K (L), respectively. All data are presented as the means ± SD of three independent experiments. **p* < 0.01.

**Supplementary Tables**

**Table S1 389 dysregulated genes detected detected in two groups**

| **Gene name** | **Gene ID** | **Regulation** | **log2 FoldChange** | | **P-value** | | **Gene biotype** | |
| --- | --- | --- | --- | --- | --- | --- | --- | --- |
| PRTN3 | ENSG00000196415 | up | 4.315885 | 0.000883 | | protein_coding | |  |
| GP2 | ENSG00000169347 | up | 4.141227 | 0.026964 | | protein_coding | |  |
| RNA5-8SN2 | ENSG00000275215 | up | 3.753491 | 0.001816 | | rRNA | |  |
| CNN2P3 | ENSG00000235832 | up | 3.735319 | 0.013512 | | processed_pseudogene | |  |
| AC011287.1 | ENSG00000229618 | up | 3.656499 | 0.009259 | | lincRNA | |  |
| AC069444.2 | ENSG00000285585 | up | 3.614983 | 0.0254 | | protein_coding | |  |
| LINC01589 | ENSG00000238120 | up | 3.413885 | 0.030802 | | antisense | |  |
| AC069277.1 | ENSG00000189229 | up | 3.393823 | 0.032605 | | lincRNA | |  |
| LCN10 | ENSG00000187922 | up | 3.207597 | 0.003004 | | protein_coding | |  |
| MIR3648-1 | ENSG00000275708 | up | 3.12994 | 0.000416 | | miRNA | |  |
| AL137002.1 | ENSG00000269125 | up | 3.121537 | 0.001341 | | antisense | |  |
| AC104695.3 | ENSG00000270640 | up | 2.990738 | 0.024644 | | sense_intronic | |  |
| AC009093.7 | ENSG00000279106 | up | 2.917183 | 0.000642 | | TEC | |  |
| AC092755.1 | ENSG00000227161 | up | 2.915225 | 0.009295 | | sense_overlapping | |  |
| FREM2 | ENSG00000150893 | up | 2.76576 | 0.005377 | | protein_coding | |  |
| SNORD1B | ENSG00000199961 | up | 2.760095 | 0.015409 | | snoRNA | |  |
| MIR6819 | ENSG00000278420 | up | 2.747884 | 0.013768 | | miRNA | |  |
| AP000753.2 | ENSG00000256789 | up | 2.72276 | 0.045048 | | antisense | |  |
| PMS2P5 | ENSG00000123965 | up | 2.710207 | 0.020487 | | unprocessed_pseudogene | |  |
| PLPPR3 | ENSG00000129951 | up | 2.697734 | 0.025246 | | protein_coding | |  |
| AC245041.1 | ENSG00000273760 | up | 2.697101 | 0.04823 | | lincRNA | |  |
| AL691482.3 | ENSG00000249007 | up | 2.696674 | 0.043027 | | sense_intronic | |  |
| SLC25A48 | ENSG00000145832 | up | 2.694441 | 0.032784 | | protein_coding | |  |
| AL358334.2 | ENSG00000258711 | up | 2.669835 | 0.029543 | | lincRNA | |  |
| AC006460.1 | ENSG00000228509 | up | 2.650517 | 0.024908 | | antisense | |  |
| ARMC4 | ENSG00000169126 | up | 2.640546 | 0.039979 | | protein_coding | |  |
| CFAP61 | ENSG00000089101 | up | 2.631545 | 0.034567 | | protein_coding | |  |
| RNU6-930P | ENSG00000212240 | up | 2.620195 | 0.0413 | | snRNA | |  |
| FTCD-AS1 | ENSG00000237338 | up | 2.615683 | 0.01143 | | antisense | |  |
| TP53AIP1 | ENSG00000120471 | up | 2.560258 | 0.049089 | | protein_coding | |  |
| AC097063.1 | ENSG00000224570 | up | 2.544122 | 0.011889 | | processed_pseudogene | |  |
| AC096633.1 | ENSG00000229220 | up | 2.535098 | 0.014862 | | processed_pseudogene | |  |
| AC126323.1 | ENSG00000166104 | up | 2.523854 | 0.031569 | | transcribed_unprocessed_pseudogene | |  |
| SNORD117 | ENSG00000201785 | up | 2.48906 | 0.028266 | | snoRNA | |  |
| AC005954.1 | ENSG00000267138 | up | 2.477033 | 0.01665 | | sense_intronic | |  |
| AC027796.5 | ENSG00000263338 | up | 2.466754 | 0.028775 | | sense_intronic | |  |
| IGBP1-AS1 | ENSG00000203588 | up | 2.407562 | 0.006043 | | antisense | |  |
| CYP4F23P | ENSG00000269516 | up | 2.387181 | 0.031791 | | transcribed_unprocessed_pseudogene | |  |
| CATIP | ENSG00000158428 | up | 2.386101 | 0.016858 | | protein_coding | |  |
| KLRC2 | ENSG00000205809 | up | 2.380068 | 0.00938 | | protein_coding | |  |
| RF00004 | ENSG00000278048 | up | 2.356965 | 0.01551 | | snRNA | |  |
| AC092634.3 | ENSG00000226581 | up | 2.337472 | 0.047916 | | lincRNA | |  |
| AC106795.2 | ENSG00000249684 | up | 2.33726 | 0.038139 | | antisense | |  |
| AC011247.2 | ENSG00000235586 | up | 2.310071 | 0.014587 | | antisense | |  |
| PNMA3 | ENSG00000183837 | up | 2.29895 | 0.001308 | | protein_coding | |  |
| LINC01977 | ENSG00000262772 | up | 2.295597 | 0.041415 | | lincRNA | |  |
| AC093382.1 | ENSG00000226276 | up | 2.288535 | 0.020708 | | lincRNA | |  |
| BGLAP | ENSG00000242252 | up | 2.254987 | 0.00138 | | protein_coding | |  |
| SLC39A2 | ENSG00000165794 | up | 2.24748 | 0.022319 | | protein_coding | |  |
| AL133319.1 | ENSG00000236378 | up | 2.23694 | 0.004533 | | lincRNA | |  |
| AL139082.1 | ENSG00000273523 | up | 2.236348 | 0.01101 | | antisense | |  |
| AC131097.4 | ENSG00000235151 | up | 2.220617 | 0.026547 | | lincRNA | |  |
| MIR324 | ENSG00000199053 | up | 2.202195 | 0.030787 | | miRNA | |  |
| AC011466.3 | ENSG00000269534 | up | 2.196532 | 0.049739 | | antisense | |  |
| ADGRA1 | ENSG00000197177 | up | 2.1774 | 0.030619 | | protein_coding | |  |
| MIR6859-1 | ENSG00000278267 | up | 2.162238 | 0.033309 | | miRNA | |  |
| AC007991.2 | ENSG00000253838 | up | 2.159744 | 0.03766 | | sense_intronic | |  |
| AL445307.1 | ENSG00000228692 | up | 2.141697 | 0.047689 | | lincRNA | |  |
| AL021937.2 | ENSG00000232218 | up | 2.138584 | 0.04894 | | unprocessed_pseudogene | |  |
| AL118511.1 | ENSG00000223393 | up | 2.10897 | 0.013597 | | antisense | |  |
| AC012435.2 | ENSG00000261775 | up | 2.076222 | 0.046891 | | lincRNA | |  |
| AC004898.1 | ENSG00000236018 | up | 2.060701 | 0.044924 | | processed_pseudogene | |  |
| AC005393.1 | ENSG00000276445 | up | 2.058453 | 0.017086 | | lincRNA | |  |
| ALAS2 | ENSG00000158578 | up | 2.040966 | 0.03465 | | protein_coding | |  |
| LINC00982 | ENSG00000177133 | up | 2.03317 | 0.006725 | | antisense | |  |
| UNC13A | ENSG00000130477 | up | 2.018435 | 0.031738 | | protein_coding | |  |
| HEATR9 | ENSG00000270379 | up | 2.011371 | 0.03018 | | protein_coding | |  |
| AL133342.1 | ENSG00000278231 | up | 2.005146 | 0.012323 | | lincRNA | |  |
| MIR126 | ENSG00000199161 | up | 1.999564 | 0.031951 | | miRNA | |  |
| KCNH2 | ENSG00000055118 | up | 1.983098 | 0.026628 | | protein_coding | |  |
| NALT1 | ENSG00000237886 | up | 1.97265 | 0.001297 | | antisense | |  |
| RPS6P25 | ENSG00000240616 | up | 1.956385 | 0.004291 | | processed_pseudogene | |  |
| AP006284.1 | ENSG00000254815 | up | 1.934329 | 8.62E-06 | | antisense | |  |
| STPG3 | ENSG00000197768 | up | 1.933778 | 0.000651 | | protein_coding | |  |
| B3GAT1 | ENSG00000109956 | up | 1.900648 | 0.001354 | | protein_coding | |  |
| AC087392.4 | ENSG00000262434 | up | 1.883359 | 0.023915 | | antisense | |  |
| TMEM262 | ENSG00000187066 | up | 1.876071 | 0.021855 | | protein_coding | |  |
| SCNN1B | ENSG00000168447 | up | 1.86207 | 0.01609 | | protein_coding | |  |
| CCDC196 | ENSG00000196553 | up | 1.859226 | 0.030909 | | protein_coding | |  |
| FOSB | ENSG00000125740 | up | 1.833618 | 0.048894 | | protein_coding | |  |
| AC009065.2 | ENSG00000259933 | up | 1.821238 | 0.010332 | | sense_overlapping | |  |
| AC023906.4 | ENSG00000259709 | up | 1.816053 | 0.003575 | | antisense | |  |
| AC012615.4 | ENSG00000267141 | up | 1.815512 | 0.033077 | | antisense | |  |
| AC135050.3 | ENSG00000260911 | up | 1.807626 | 0.015999 | | lincRNA | |  |
| AP006285.1 | ENSG00000227306 | up | 1.803101 | 0.025183 | | lincRNA | |  |
| MT1M | ENSG00000205364 | up | 1.797512 | 0.030477 | | protein_coding | |  |
| AC011747.1 | ENSG00000231083 | up | 1.797386 | 0.042862 | | lincRNA | |  |
| PRSS30P | ENSG00000172460 | up | 1.768519 | 0.009729 | | transcribed_unitary_pseudogene | |  |
| GALNT8 | ENSG00000130035 | up | 1.765989 | 0.034113 | | protein_coding | |  |
| AL135999.1 | ENSG00000258727 | up | 1.764572 | 0.019128 | | antisense | |  |
| AL031963.2 | ENSG00000229282 | up | 1.757927 | 0.043643 | | lincRNA | |  |
| AC090510.2 | ENSG00000274403 | up | 1.734313 | 0.008596 | | antisense | |  |
| AC009093.2 | ENSG00000260517 | up | 1.706373 | 0.001061 | | lincRNA | |  |
| FAM57B | ENSG00000149926 | up | 1.70467 | 0.047675 | | protein_coding | |  |
| AC100803.3 | ENSG00000271959 | up | 1.699795 | 0.017643 | | antisense | |  |
| AP006621.4 | ENSG00000269915 | up | 1.695775 | 0.033806 | | antisense | |  |
| CBSL | ENSG00000274276 | up | 1.695772 | 0.027429 | | protein_coding | |  |
| AC025048.4 | ENSG00000267416 | up | 1.693101 | 0.019275 | | lincRNA | |  |
| AL645608.7 | ENSG00000272512 | up | 1.690365 | 0.01255 | | lincRNA | |  |
| AC093249.2 | ENSG00000260167 | up | 1.661051 | 0.046684 | | antisense | |  |
| VPS9D1-AS1 | ENSG00000261373 | up | 1.658464 | 0.001666 | | antisense | |  |
| ELAVL4 | ENSG00000162374 | up | 1.646595 | 0.033759 | | protein_coding | |  |
| AC138230.1 | ENSG00000255237 | up | 1.630528 | 0.032241 | | antisense | |  |
| ASPG | ENSG00000166183 | up | 1.62568 | 0.024727 | | protein_coding | |  |
| SNORA73B | ENSG00000200087 | up | 1.619239 | 0.024455 | | snoRNA | |  |
| TBC1D3G | ENSG00000260287 | up | 1.60324 | 0.045614 | | protein_coding | |  |
| AC004381.1 | ENSG00000260510 | up | 1.598816 | 0.049982 | | antisense | |  |
| CTD-3080P12.3 | ENSG00000249201 | up | 1.591593 | 0.033154 | | antisense | |  |
| USP43 | ENSG00000154914 | up | 1.590245 | 0.007747 | | protein_coding | |  |
| PRDM16 | ENSG00000142611 | up | 1.582254 | 0.022842 | | protein_coding | |  |
| AL391069.3 | ENSG00000237976 | up | 1.581732 | 0.039611 | | antisense | |  |
| AC009949.1 | ENSG00000279964 | up | 1.576491 | 0.047282 | | TEC | |  |
| LRRC19 | ENSG00000184434 | up | 1.556129 | 0.011177 | | protein_coding | |  |
| DKK4 | ENSG00000104371 | up | 1.549021 | 0.0317 | | protein_coding | |  |
| GHRHR | ENSG00000106128 | up | 1.535194 | 0.037457 | | protein_coding | |  |
| AC011448.1 | ENSG00000258674 | up | 1.529071 | 0.045977 | | protein_coding | |  |
| PADI1 | ENSG00000142623 | up | 1.514232 | 0.0446 | | protein_coding | |  |
| MEG3 | ENSG00000214548 | up | 1.500918 | 0.031966 | | lincRNA | |  |
| GLYCTK-AS1 | ENSG00000242797 | up | 1.498702 | 0.004141 | | processed_transcript | |  |
| LINC01238 | ENSG00000237940 | up | 1.496364 | 0.001771 | | lincRNA | |  |
| LINC01786 | ENSG00000230415 | up | 1.487021 | 0.001511 | | lincRNA | |  |
| EGFL8 | ENSG00000241404 | up | 1.463894 | 0.02404 | | protein_coding | |  |
| NR4A3 | ENSG00000119508 | up | 1.454634 | 0.039961 | | protein_coding | |  |
| AC007663.1 | ENSG00000161132 | up | 1.451687 | 0.023758 | | unprocessed_pseudogene | |  |
| RXRG | ENSG00000143171 | up | 1.44503 | 0.020787 | | protein_coding | |  |
| MYCBPAP | ENSG00000136449 | up | 1.442462 | 0.017525 | | protein_coding | |  |
| STPG3-AS1 | ENSG00000275549 | up | 1.440294 | 0.023686 | | antisense | |  |
| AC110285.2 | ENSG00000262877 | up | 1.438244 | 0.03597 | | lincRNA | |  |
| SGSM3 | ENSG00000100359 | up | 1.423091 | 0.001444 | | protein_coding | |  |
| LHX2 | ENSG00000106689 | up | 1.422178 | 0.034856 | | protein_coding | |  |
| AC022075.1 | ENSG00000245648 | up | 1.420002 | 0.047199 | | antisense | |  |
| AC092053.3 | ENSG00000284669 | up | 1.417056 | 0.033923 | | antisense | |  |
| RND2 | ENSG00000108830 | up | 1.415569 | 0.037153 | | protein_coding | |  |
| AC020907.5 | ENSG00000279619 | up | 1.384069 | 0.046852 | | TEC | |  |
| MT1DP | ENSG00000205361 | up | 1.381464 | 0.038957 | | transcribed_unprocessed_pseudogene | |  |
| AL139385.1 | ENSG00000275880 | up | 1.351979 | 0.040282 | | antisense | |  |
| AC016722.2 | ENSG00000228925 | up | 1.351224 | 0.025714 | | antisense | |  |
| AL137060.1 | ENSG00000274270 | up | 1.33653 | 0.012588 | | sense_intronic | |  |
| SH2D6 | ENSG00000152292 | up | 1.325421 | 0.037919 | | protein_coding | |  |
| LINC-PINT | ENSG00000226380 | up | 1.323264 | 0.009932 | | lincRNA | |  |
| NR4A1 | ENSG00000123358 | up | 1.322293 | 0.033158 | | protein_coding | |  |
| CXCL2 | ENSG00000081041 | up | 1.31876 | 0.030303 | | protein_coding | |  |
| AC007376.2 | ENSG00000258569 | up | 1.313838 | 0.048953 | | lincRNA | |  |
| AC006128.1 | ENSG00000279716 | up | 1.310954 | 0.017263 | | TEC | |  |
| AC116407.4 | ENSG00000280033 | up | 1.300544 | 0.026469 | | TEC | |  |
| LINC01451 | ENSG00000279141 | up | 1.282729 | 0.034529 | | lincRNA | |  |
| AL162431.2 | ENSG00000243155 | up | 1.28125 | 0.02146 | | antisense | |  |
| AC006254.1 | ENSG00000243696 | up | 1.28102 | 0.035435 | | protein_coding | |  |
| CCNJL | ENSG00000135083 | up | 1.270353 | 0.012253 | | protein_coding | |  |
| U73166.1 | ENSG00000230454 | up | 1.262316 | 0.017693 | | lincRNA | |  |
| AC099489.1 | ENSG00000188897 | up | 1.258847 | 0.040338 | | protein_coding | |  |
| AC027796.4 | ENSG00000262903 | up | 1.25795 | 0.014517 | | antisense | |  |
| RF00019 | ENSG00000252759 | up | 1.24511 | 0.042796 | | misc_RNA | |  |
| AC008735.2 | ENSG00000267523 | up | 1.242849 | 0.002922 | | antisense | |  |
| STX16-NPEPL1 | ENSG00000254995 | up | 1.239581 | 0.028848 | | protein_coding | |  |
| LINC00106 | ENSG00000236871 | up | 1.231467 | 0.005982 | | lincRNA | |  |
| CDK11A | ENSG00000008128 | up | 1.231238 | 0.004012 | | protein_coding | |  |
| AC138956.2 | ENSG00000251414 | up | 1.224016 | 0.007135 | | antisense | |  |
| NR4A2 | ENSG00000153234 | up | 1.215798 | 0.027349 | | protein_coding | |  |
| AC131009.4 | ENSG00000279283 | up | 1.213005 | 0.04085 | | TEC | |  |
| AC110015.1 | ENSG00000227279 | up | 1.208113 | 0.042951 | | antisense | |  |
| CHKB | ENSG00000100288 | up | 1.207903 | 0.027712 | | protein_coding | |  |
| ARL6IP4 | ENSG00000182196 | up | 1.206128 | 0.023982 | | protein_coding | |  |
| C1orf220 | ENSG00000213057 | up | 1.189613 | 0.004962 | | lincRNA | |  |
| AL451007.1 | ENSG00000232059 | up | 1.183267 | 0.04241 | | processed_pseudogene | |  |
| AC114947.2 | ENSG00000261604 | up | 1.172898 | 0.041203 | | antisense | |  |
| AC132872.3 | ENSG00000275888 | up | 1.168017 | 0.001128 | | antisense | |  |
| DCXR | ENSG00000169738 | up | 1.164147 | 0.018712 | | protein_coding | |  |
| AC005519.1 | ENSG00000258559 | up | 1.162593 | 0.010402 | | sense_overlapping | |  |
| ZDHHC11B | ENSG00000206077 | up | 1.152397 | 0.00748 | | protein_coding | |  |
| ARG2 | ENSG00000081181 | up | 1.150799 | 0.032105 | | protein_coding | |  |
| AP001107.1 | ENSG00000245156 | up | 1.149229 | 0.006738 | | lincRNA | |  |
| AC020916.1 | ENSG00000267519 | up | 1.147818 | 0.022103 | | antisense | |  |
| SLC39A4 | ENSG00000147804 | up | 1.142462 | 0.035946 | | protein_coding | |  |
| TEN1-CDK3 | ENSG00000261408 | up | 1.13694 | 0.023853 | | protein_coding | |  |
| AC233968.1 | ENSG00000274615 | up | 1.12419 | 0.033923 | | unprocessed_pseudogene | |  |
| LINC02449 | ENSG00000215241 | up | 1.109521 | 0.048559 | | lincRNA | |  |
| GRIN3B | ENSG00000116032 | up | 1.105908 | 0.018886 | | protein_coding | |  |
| SLCO4A1 | ENSG00000101187 | up | 1.104344 | 0.041741 | | protein_coding | |  |
| NOCT | ENSG00000151014 | up | 1.076323 | 0.015499 | | protein_coding | |  |
| AC137767.1 | ENSG00000256092 | up | 1.067576 | 0.017711 | | lincRNA | |  |
| LCN12 | ENSG00000184925 | up | 1.064783 | 0.013647 | | protein_coding | |  |
| AL603839.2 | ENSG00000238186 | up | 1.064337 | 0.043243 | | antisense | |  |
| LINC00894 | ENSG00000235703 | up | 1.061286 | 0.028429 | | antisense | |  |
| LRRC37A7P | ENSG00000265158 | up | 1.056849 | 0.034693 | | transcribed_processed_pseudogene | |  |
| CDK3 | ENSG00000250506 | up | 1.056756 | 0.025086 | | protein_coding | |  |
| AL137145.2 | ENSG00000215244 | up | 1.048815 | 0.045186 | | lincRNA | |  |
| WDR97 | ENSG00000179698 | up | 1.043069 | 0.005288 | | protein_coding | |  |
| AL139287.1 | ENSG00000240731 | up | 1.027034 | 0.001206 | | sense_intronic | |  |
| AC093525.8 | ENSG00000279520 | up | 1.020685 | 0.000345 | | TEC | |  |
| AC012615.6 | ENSG00000267244 | up | 1.018416 | 0.014417 | | processed_transcript | |  |
| DUSP1 | ENSG00000120129 | up | 1.013258 | 0.0478 | | protein_coding | |  |
| CSRNP1 | ENSG00000144655 | up | 1.009012 | 0.027817 | | protein_coding | |  |
| AL590764.1 | ENSG00000228427 | up | 1.003208 | 0.022694 | | antisense | |  |
| AC010203.2 | ENSG00000257696 | up | 0.999543 | 0.005059 | | antisense | |  |
| AC141557.1 | ENSG00000256673 | up | 0.997612 | 0.021483 | | unprocessed_pseudogene | |  |
| MIR5581 | ENSG00000263675 | up | 0.992287 | 0.02813 | | miRNA | |  |
| LENG8-AS1 | ENSG00000226696 | up | 0.991156 | 0.026492 | | antisense | |  |
| PLA2G6 | ENSG00000184381 | up | 0.990166 | 0.031773 | | protein_coding | |  |
| AL354696.2 | ENSG00000278390 | up | 0.97317 | 0.046397 | | antisense | |  |
| PHBP9 | ENSG00000230224 | up | 0.973133 | 0.023543 | | processed_pseudogene | |  |
| MZF1-AS1 | ENSG00000267858 | up | 0.966656 | 0.025349 | | antisense | |  |
| LINC01089 | ENSG00000212694 | up | 0.965378 | 0.007398 | | lincRNA | |  |
| MIR4783 | ENSG00000264075 | up | 0.96121 | 0.04681 | | miRNA | |  |
| C17orf82 | ENSG00000187013 | up | 0.958322 | 0.037533 | | lincRNA | |  |
| KIZ-AS1 | ENSG00000232712 | up | 0.956002 | 0.037562 | | antisense | |  |
| PI4KAP1 | ENSG00000274602 | up | 0.95382 | 0.049271 | | transcribed_unprocessed_pseudogene | |  |
| AC084018.1 | ENSG00000272849 | up | 0.953742 | 0.023779 | | lincRNA | |  |
| AL591845.1 | ENSG00000116883 | up | 0.942641 | 0.017407 | | antisense | |  |
| PPT2-EGFL8 | ENSG00000258388 | up | 0.941087 | 0.039785 | | protein_coding | |  |
| AC009962.1 | ENSG00000260742 | up | 0.940968 | 0.034379 | | antisense | |  |
| Z83840.1 | ENSG00000285531 | up | 0.93943 | 0.030052 | | processed_pseudogene | |  |
| AC145207.8 | ENSG00000264769 | up | 0.938042 | 0.040237 | | antisense | |  |
| AC084125.2 | ENSG00000255182 | up | 0.935405 | 0.004102 | | processed_transcript | |  |
| AC008764.6 | ENSG00000269399 | up | 0.929436 | 0.021971 | | lincRNA | |  |
| LINC00893 | ENSG00000241769 | up | 0.92205 | 0.037277 | | antisense | |  |
| MIR3936 | ENSG00000263597 | up | 0.920369 | 0.046066 | | miRNA | |  |
| AC244197.3 | ENSG00000241489 | up | 0.919795 | 0.042462 | | protein_coding | |  |
| AP001062.1 | ENSG00000184441 | up | 0.91853 | 0.017373 | | antisense | |  |
| SIK1B | ENSG00000275993 | up | 0.917451 | 0.031915 | | protein_coding | |  |
| GPS2 | ENSG00000132522 | up | 0.913457 | 0.033519 | | protein_coding | |  |
| CRACR2B | ENSG00000177685 | up | 0.900444 | 0.0304 | | protein_coding | |  |
| AL022328.3 | ENSG00000273188 | up | 0.897416 | 0.017802 | | antisense | |  |
| AP006621.5 | ENSG00000279672 | up | 0.89407 | 0.005702 | | TEC | |  |
| BRICD5 | ENSG00000182685 | up | 0.891805 | 0.037466 | | protein_coding | |  |
| AC009065.8 | ENSG00000261663 | up | 0.891304 | 0.003719 | | antisense | |  |
| KIAA0895L | ENSG00000196123 | up | 0.888808 | 0.006977 | | protein_coding | |  |
| SCNN1D | ENSG00000162572 | up | 0.887095 | 0.016252 | | protein_coding | |  |
| AC145285.6 | ENSG00000275807 | up | 0.882036 | 0.027792 | | antisense | |  |
| LINC00482 | ENSG00000185168 | up | 0.878805 | 0.033341 | | lincRNA | |  |
| HDAC10 | ENSG00000100429 | up | 0.87813 | 0.029804 | | protein_coding | |  |
| ZCWPW2 | ENSG00000206559 | up | 0.876938 | 0.036249 | | protein_coding | |  |
| HBEGF | ENSG00000113070 | up | 0.871863 | 0.049738 | | protein_coding | |  |
| EIF4A1 | ENSG00000161960 | up | 0.871064 | 0.042183 | | protein_coding | |  |
| LPIN3 | ENSG00000132793 | up | 0.861987 | 0.002195 | | protein_coding | |  |
| AC018665.1 | ENSG00000279382 | up | 0.853997 | 0.02139 | | TEC | |  |
| PTOV1-AS2 | ENSG00000269352 | up | 0.851489 | 0.024964 | | antisense | |  |
| PDXDC2P-NPIPB14P | ENSG00000196696 | up | 0.84883 | 0.008734 | | processed_transcript | |  |
| CLTCL1 | ENSG00000070371 | up | 0.842877 | 0.021023 | | protein_coding | |  |
| AC004890.2 | ENSG00000244560 | up | 0.83074 | 0.031195 | | transcribed_unprocessed_pseudogene | |  |
| D2HGDH | ENSG00000180902 | up | 0.826764 | 0.01254 | | protein_coding | |  |
| USP54 | ENSG00000166348 | up | 0.805812 | 0.030044 | | protein_coding | |  |
| RNF207 | ENSG00000158286 | up | 0.79264 | 0.004657 | | protein_coding | |  |
| KIFC2 | ENSG00000167702 | up | 0.791349 | 0.036434 | | protein_coding | |  |
| LENG8 | ENSG00000167615 | up | 0.789765 | 0.027995 | | protein_coding | |  |
| JMJD7-PLA2G4B | ENSG00000168970 | up | 0.786488 | 0.031451 | | protein_coding | |  |
| CDK5RAP3 | ENSG00000108465 | up | 0.785106 | 0.02302 | | protein_coding | |  |
| DHPS | ENSG00000095059 | up | 0.779446 | 0.0407 | | protein_coding | |  |
| STAG3L1 | ENSG00000205583 | up | 0.767337 | 0.016699 | | transcribed_unprocessed_pseudogene | |  |
| STAG3L5P | ENSG00000242294 | up | 0.76173 | 0.007885 | | transcribed_unprocessed_pseudogene | |  |
| PHOSPHO1 | ENSG00000173868 | up | 0.749293 | 0.039997 | | protein_coding | |  |
| AC005225.4 | ENSG00000279026 | up | 0.746041 | 0.035346 | | TEC | |  |
| ZMYND15 | ENSG00000141497 | up | 0.745248 | 0.045467 | | protein_coding | |  |
| AC022211.2 | ENSG00000263843 | up | 0.732065 | 0.049682 | | antisense | |  |
| TOR1AIP2 | ENSG00000169905 | up | 0.726931 | 0.04452 | | protein_coding | |  |
| AC069281.2 | ENSG00000274272 | up | 0.72428 | 0.010679 | | processed_transcript | |  |
| ATG16L2 | ENSG00000168010 | up | 0.722236 | 0.011178 | | protein_coding | |  |
| NAALAD2 | ENSG00000077616 | up | 0.718367 | 0.041857 | | protein_coding | |  |
| SLC9A3 | ENSG00000066230 | up | 0.715827 | 0.037183 | | protein_coding | |  |
| MMP19 | ENSG00000123342 | up | 0.714917 | 0.049667 | | protein_coding | |  |
| PIDD1 | ENSG00000177595 | up | 0.710595 | 0.006711 | | protein_coding | |  |
| ATAD3B | ENSG00000160072 | up | 0.708327 | 0.040226 | | protein_coding | |  |
| PILRB | ENSG00000121716 | up | 0.697725 | 0.023337 | | protein_coding | |  |
| NSUN5P2 | ENSG00000106133 | up | 0.696152 | 0.047551 | | transcribed_unprocessed_pseudogene | |  |
| PPP1R26-AS1 | ENSG00000225361 | up | 0.688469 | 0.04381 | | antisense | |  |
| ZNF451-AS1 | ENSG00000226803 | up | 0.675901 | 0.040468 | | antisense | |  |
| DNHD1 | ENSG00000179532 | up | 0.674717 | 0.004903 | | protein_coding | |  |
| SRRM2 | ENSG00000167978 | up | 0.665739 | 0.021289 | | protein_coding | |  |
| MRNIP | ENSG00000161010 | up | 0.661958 | 0.030794 | | protein_coding | |  |
| TEPSIN | ENSG00000167302 | up | 0.661494 | 0.02625 | | protein_coding | |  |
| PABPN1 | ENSG00000100836 | up | 0.659141 | 0.010847 | | protein_coding | |  |
| AL353622.1 | ENSG00000270605 | up | 0.657209 | 0.024549 | | antisense | |  |
| U2AF1L4 | ENSG00000161265 | up | 0.649031 | 0.038159 | | protein_coding | |  |
| CHRD | ENSG00000090539 | up | 0.645155 | 0.047402 | | protein_coding | |  |
| ZNF276 | ENSG00000158805 | up | 0.643185 | 0.048669 | | protein_coding | |  |
| AL731566.1 | ENSG00000273891 | up | 0.637664 | 0.031563 | | antisense | |  |
| KLHL17 | ENSG00000187961 | up | 0.624535 | 0.031365 | | protein_coding | |  |
| TUBGCP6 | ENSG00000128159 | up | 0.618307 | 0.013362 | | protein_coding | |  |
| AL049840.1 | ENSG00000246451 | up | 0.59758 | 0.02765 | | antisense | |  |
| FBXL8 | ENSG00000135722 | up | 0.595865 | 0.014089 | | protein_coding | |  |
| NDUFB1 | ENSG00000183648 | up | 0.593493 | 0.009161 | | protein_coding | |  |
| AKAP8L | ENSG00000011243 | up | 0.575384 | 0.029068 | | protein_coding | |  |
| SNHG15 | ENSG00000232956 | up | 0.569416 | 0.047484 | | lincRNA | |  |
| FAM193B | ENSG00000146067 | up | 0.566258 | 0.038897 | | protein_coding | |  |
| ACAP3 | ENSG00000131584 | up | 0.544976 | 0.037604 | | protein_coding | |  |
| MBD6 | ENSG00000166987 | up | 0.507621 | 0.033192 | | protein_coding | |  |
| UVSSA | ENSG00000163945 | up | 0.48526 | 0.046823 | | protein_coding | |  |
| TMEM198B | ENSG00000182796 | up | 0.482311 | 0.029675 | | transcribed_unitary_pseudogene | |  |
| TAF1C | ENSG00000103168 | up | 0.47302 | 0.026819 | | protein_coding | |  |
| LMBR1L | ENSG00000139636 | up | 0.439959 | 0.03514 | | protein_coding | |  |
| CPSF7 | ENSG00000149532 | up | 0.433189 | 0.042089 | | protein_coding | |  |
| UBE2V1 | ENSG00000244687 | up | 0.424897 | 0.047293 | | protein_coding | |  |
| PICK1 | ENSG00000100151 | up | 0.405838 | 0.020014 | | protein_coding | |  |
| CENPT | ENSG00000102901 | up | 0.400315 | 0.038887 | | protein_coding | |  |
| SPG7 | ENSG00000197912 | up | 0.381488 | 0.049543 | | protein_coding | |  |
| ZSWIM8 | ENSG00000214655 | up | 0.355322 | 0.042617 | | protein_coding | |  |
| ABCD4 | ENSG00000119688 | up | 0.33545 | 0.047896 | | protein_coding | |  |
| TRPC4AP | ENSG00000100991 | up | 0.260277 | 0.039858 | | protein_coding | |  |
| DMBT1 | ENSG00000187908 | down | -4.11116 | 0.001293 | | protein_coding | |  |
| MYBPH | ENSG00000133055 | down | -3.41076 | 0.012576 | | protein_coding | |  |
| AL133467.4 | ENSG00000270038 | down | -3.31139 | 0.025724 | | lincRNA | |  |
| LINC00640 | ENSG00000258479 | down | -3.23535 | 0.011491 | | lincRNA | |  |
| AC010368.1 | ENSG00000250886 | down | -2.9743 | 0.022442 | | processed_pseudogene | |  |
| AC092650.1 | ENSG00000230773 | down | -2.93022 | 0.036906 | | lincRNA | |  |
| MUC5B | ENSG00000117983 | down | -2.82042 | 0.001883 | | protein_coding | |  |
| SOX21 | ENSG00000125285 | down | -2.77307 | 0.037873 | | protein_coding | |  |
| AC138035.2 | ENSG00000250765 | down | -2.7498 | 0.007788 | | lincRNA | |  |
| AC114781.1 | ENSG00000248401 | down | -2.73998 | 0.048338 | | processed_pseudogene | |  |
| HCP5B | ENSG00000281831 | down | -2.63035 | 0.01061 | | lincRNA | |  |
| OVOL1-AS1 | ENSG00000255120 | down | -2.61236 | 0.028239 | | antisense | |  |
| NUTM2F | ENSG00000130950 | down | -2.56322 | 0.017844 | | protein_coding | |  |
| RHCG | ENSG00000140519 | down | -2.55692 | 0.003741 | | protein_coding | |  |
| AL117328.2 | ENSG00000279494 | down | -2.53688 | 0.033626 | | lincRNA | |  |
| XXYLT1-AS2 | ENSG00000230266 | down | -2.5076 | 0.043061 | | antisense | |  |
| ISCA1P4 | ENSG00000259405 | down | -2.49538 | 0.022137 | | processed_pseudogene | |  |
| LINC01819 | ENSG00000231826 | down | -2.47946 | 0.018404 | | lincRNA | |  |
| RPS15AP16 | ENSG00000239483 | down | -2.476 | 0.037908 | | processed_pseudogene | |  |
| MT-TT | ENSG00000210195 | down | -2.47591 | 0.011882 | | Mt_tRNA | |  |
| AC113383.1 | ENSG00000250320 | down | -2.45722 | 0.038186 | | antisense | |  |
| AC106796.1 | ENSG00000263237 | down | -2.31569 | 0.009479 | | antisense | |  |
| UBTFL6 | ENSG00000228970 | down | -2.30624 | 0.026318 | | unprocessed_pseudogene | |  |
| RPSAP47 | ENSG00000188856 | down | -2.25578 | 0.037333 | | processed_pseudogene | |  |
| POU1F1 | ENSG00000064835 | down | -2.2397 | 0.047896 | | protein_coding | |  |
| AL157700.1 | ENSG00000260118 | down | -2.23636 | 0.030492 | | lincRNA | |  |
| ELAVL2 | ENSG00000107105 | down | -2.23464 | 0.042743 | | protein_coding | |  |
| PCDHA1 | ENSG00000204970 | down | -2.17966 | 0.036748 | | protein_coding | |  |
| AL031777.1 | ENSG00000217275 | down | -2.15039 | 0.031259 | | processed_pseudogene | |  |
| RF00019 | ENSG00000207342 | down | -2.13304 | 0.02428 | | misc_RNA | |  |
| AL031666.3 | ENSG00000273451 | down | -2.09411 | 0.025523 | | sense_intronic | |  |
| AL603910.1 | ENSG00000223821 | down | -2.06193 | 0.02784 | | antisense | |  |
| ZRANB2-AS1 | ENSG00000235079 | down | -2.00739 | 0.017528 | | antisense | |  |
| AC020612.1 | ENSG00000257243 | down | -1.98378 | 0.0232 | | processed_pseudogene | |  |
| CYP11A1 | ENSG00000140459 | down | -1.97964 | 0.049599 | | protein_coding | |  |
| AC234781.1 | ENSG00000224216 | down | -1.8651 | 0.03849 | | antisense | |  |
| AC022973.3 | ENSG00000254263 | down | -1.84268 | 0.035691 | | lincRNA | |  |
| AL049794.1 | ENSG00000273998 | down | -1.8367 | 0.041804 | | lincRNA | |  |
| AP001636.2 | ENSG00000254877 | down | -1.81862 | 0.045156 | | processed_pseudogene | |  |
| GUCY2EP | ENSG00000204529 | down | -1.79485 | 0.029957 | | transcribed_unprocessed_pseudogene | |  |
| AL391650.1 | ENSG00000236782 | down | -1.71257 | 0.045774 | | protein_coding | |  |
| MLIP-AS1 | ENSG00000235050 | down | -1.67283 | 0.031799 | | antisense | |  |
| ADAM21 | ENSG00000139985 | down | -1.67018 | 0.045639 | | protein_coding | |  |
| PCSK1 | ENSG00000175426 | down | -1.64997 | 0.010681 | | protein_coding | |  |
| ACTA1 | ENSG00000143632 | down | -1.64617 | 0.021581 | | protein_coding | |  |
| AL139094.1 | ENSG00000220685 | down | -1.55985 | 0.039038 | | processed_pseudogene | |  |
| KLHDC7B | ENSG00000130487 | down | -1.55948 | 0.012417 | | protein_coding | |  |
| MT-TY | ENSG00000210144 | down | -1.55718 | 0.013075 | | Mt_tRNA | |  |
| ZNF385D | ENSG00000151789 | down | -1.51967 | 0.047705 | | protein_coding | |  |
| AC046143.2 | ENSG00000272707 | down | -1.47096 | 0.043352 | | lincRNA | |  |
| SPOCD1 | ENSG00000134668 | down | -1.45037 | 0.041834 | | protein_coding | |  |
| KRTAP5-6 | ENSG00000205864 | down | -1.39211 | 0.036778 | | protein_coding | |  |
| APOD | ENSG00000189058 | down | -1.38025 | 0.038251 | | protein_coding | |  |
| KLHL7-DT | ENSG00000230658 | down | -1.37743 | 0.018048 | | lincRNA | |  |
| F13A1 | ENSG00000124491 | down | -1.37096 | 0.014056 | | protein_coding | |  |
| RPL4P6 | ENSG00000230071 | down | -1.369 | 0.03631 | | processed_pseudogene | |  |
| ADORA1 | ENSG00000163485 | down | -1.32542 | 0.0313 | | protein_coding | |  |
| SLC5A10 | ENSG00000154025 | down | -1.30915 | 0.019296 | | protein_coding | |  |
| SV2C | ENSG00000122012 | down | -1.30089 | 0.045578 | | protein_coding | |  |
| RSL24D1P6 | ENSG00000224452 | down | -1.28825 | 0.049603 | | processed_pseudogene | |  |
| ENKUR | ENSG00000151023 | down | -1.20447 | 0.035135 | | protein_coding | |  |
| AC008035.1 | ENSG00000272369 | down | -1.19479 | 0.043996 | | lincRNA | |  |
| PDGFRL | ENSG00000104213 | down | -1.13875 | 0.012421 | | protein_coding | |  |
| AC010343.1 | ENSG00000240376 | down | -1.09031 | 0.035774 | | processed_pseudogene | |  |
| FGF12 | ENSG00000114279 | down | -1.02382 | 0.041459 | | protein_coding | |  |
| AC105277.1 | ENSG00000232453 | down | -1.00158 | 0.046803 | | lincRNA | |  |
| UBAC2-AS1 | ENSG00000228889 | down | -0.95192 | 0.040343 | | lincRNA | |  |
| AL109918.1 | ENSG00000216775 | down | -0.86447 | 0.038348 | | transcribed_unprocessed_pseudogene | |  |
| AL136298.3 | ENSG00000259086 | down | -0.81282 | 0.027327 | | processed_pseudogene | |  |
| MIR34AHG | ENSG00000228526 | down | -0.80394 | 0.001529 | | lincRNA | |  |
| AL157394.1 | ENSG00000261438 | down | -0.80095 | 0.048188 | | sense_overlapping | |  |
| AL136981.2 | ENSG00000275318 | down | -0.75714 | 0.046971 | | unprocessed_pseudogene | |  |
| ABCA3 | ENSG00000167972 | down | -0.73243 | 0.03086 | | protein_coding | |  |
| ANKRD18EP | ENSG00000217165 | down | -0.69118 | 0.014468 | | processed_pseudogene | |  |
| AL117332.1 | ENSG00000275457 | down | -0.60057 | 0.049517 | | antisense | |  |
| ZNF416 | ENSG00000083817 | down | -0.57761 | 0.010637 | | protein_coding | |  |
| AC122718.1 | ENSG00000250461 | down | -0.55082 | 0.035049 | | processed_pseudogene | |  |
| ZNF112 | ENSG00000062370 | down | -0.53167 | 0.026074 | | protein_coding | |  |
| ZRANB3 | ENSG00000121988 | down | -0.51759 | 0.029878 | | protein_coding | |  |
| ZNF543 | ENSG00000178229 | down | -0.44367 | 0.018733 | | protein_coding | |  |
| PRPF18 | ENSG00000165630 | down | -0.39517 | 0.044719 | | protein_coding | |  |
| ZNF627 | ENSG00000198551 | down | -0.37752 | 0.047946 | | protein_coding | |  |
| ZNF12 | ENSG00000164631 | down | -0.34108 | 0.018627 | | protein_coding | |  |
| UBN2 | ENSG00000157741 | down | -0.3255 | 0.046451 | | protein_coding | |  |
| RNMT | ENSG00000101654 | down | -0.32112 | 0.033555 | | protein_coding | |  |
| KIAA1143 | ENSG00000163807 | down | -0.31124 | 0.039701 | | protein_coding | |  |
| ZNF182 | ENSG00000147118 | down | -0.30452 | 0.032412 | | protein_coding | |  |
| ZNF766 | ENSG00000196214 | down | -0.28096 | 0.047726 | | protein_coding | |  |
| ASCC3 | ENSG00000112249 | down | -0.27874 | 0.037467 | | protein_coding | |  |
| DBR1 | ENSG00000138231 | down | -0.25412 | 0.044394 | | protein_coding | |  |
| ZNF146 | ENSG00000167635 | down | -0.25055 | 0.035704 | | protein_coding | |  |

**Table S2 20 dysregulated proteins detected in two groups**

| **Protein IDs** | **Gene Name** | **Peptides** | **Mol. weight [kDa]** | **Regulation** | **log2 FoldChange** | **P-value** |
| --- | --- | --- | --- | --- | --- | --- |
| P49913 | CAMP | 6 | 19.301 | UP | 3.9808 | 0.0388 |
| P14780 | MMP9 | 9 | 78.457 | UP | 3.5363 | 0.0078 |
| P80511 | S100A12 | 3 | 10.575 | UP | 3.0851 | 0.0356 |
| P06702 | S100A9 | 7 | 13.242 | UP | 2.6283 | 0.0402 |
| P05109 | S100A8 | 8 | 10.834 | UP | 2.4884 | 0.0336 |
| Q9NW64 | RBM22 | 5 | 46.895 | UP | 2.0933 | 0.0341 |
| P02788 | LTF | 27 | 78.181 | UP | 1.9788 | 0.0343 |
| P24158 | PRTN3 | 5 | 27.807 | UP | 1.9009 | 0.0392 |
| Q8N668 | COMMD1 | 5 | 21.178 | UP | 1.8555 | 0.0019 |
| Q5ZPR3 | CD276 | 6 | 57.235 | UP | 1.8114 | 0.0431 |
| P11802 | CDK4 | 6 | 33.729 | UP | 1.7969 | 0.0456 |
| P51690 | ARSL | 12 | 65.668 | UP | 1.7749 | 0.0465 |
| Q765P7 | MTSS2 | 11 | 79.928 | UP | 1.7374 | 0.0293 |
| O95563 | MPC2 | 4 | 14.279 | UP | 1.7234 | 0.0235 |
| P62877 | RBX1 | 3 | 12.274 | DOWN | 0.6568 | 0.0179 |
| Q8N490 | PNKD | 6 | 42.875 | DOWN | 0.6322 | 0.0059 |
| Q9HAC7 | SUGCT | 9 | 48.461 | DOWN | 0.5247 | 0.0259 |
| Q5JTZ9 | AARS2 | 26 | 107.34 | DOWN | 0.4404 | 0.0485 |
| Q9Y6X4 | FAM169A | 9 | 74.954 | DOWN | 0.3982 | 0.0127 |
| Q13402;Q6PIF6 | MYO7A | 22 | 254.39 | DOWN | 0.3706 | 0.0408 |

**Table S3 Proteins with significantly differential expression in subcellular location**

| **Locations** | **Name** | **Diff** | **DiffSeqs** | **DiffGenes** |
| --- | --- | --- | --- | --- |
| cyto | cytosol | 6 | P11802;Q8N668;Q9NW64;Q13402;  P80511;P06702 | CDK4,COMMD1,RBM22,MYO7A,S100A12,S100A9 |
| extr | extracellular | 5 | P49913;P02788;P24158;P62877;  P14780 | CAMP,LTF,PRTN3,RBX1,MMP9 |
| mito | mitochondria | 5 | Q5JTZ9;P05109;O95563;Q8N490;  Q9HAC7 | AARS2,S100A8,MPC2,PNKD,SUGCT |
| nucl | nucleus | 2 | Q9Y6X4;Q765P7 | FAM169A,MTSS2 |
| plas | plasma membrane | 2 | P51690;Q5ZPR3 | ARSL,CD276 |

**Table S4 Transcription factor associated proteins**

| **TF_family** | **#Diff** | **DiffSeqs** | **DiffGenes** |
| --- | --- | --- | --- |
| bHLH | 4 | Q9UH92;P61244;Q15596;Q15853 | MLX,MAX,NCOA2,USF2 |
| CTF/NFI | 1 | Q12857 | NFIA |
| Ets | 1 | Q01543 | FLI1 |
| GCFC | 1 | Q9Y5B6 | PAXBP1 |
| HMG_box | 1 | Q86U86 | PBRM1 |
| HSF_DNA-bind | 1 | Q00613 | HSF1 |
| IRF | 1 | Q13568 | IRF5 |
| LRRFIP | 1 | Q9UHR5 | SAP30BP |
| Myb_DNA-bd | 1 | Q15554 | TERF2 |
| RHD | 1 | Q13469 | NFATC2 |
| THAP | 1 | Q8WY91 | THAP4 |
| ZBTB | 1 | Q4G0X4 | KCTD21 |
| zf-BED | 1 | P61513 | RPL37A |
| zf-C2H2 | 1 | Q9BWE0 | REPIN1 |

**Table S5 Integrated data of transcriptome and proteome for GO and KEGG pathway enrichment analysis**

| **Gene Set Name** | **Description** | **Genes in Gene Set** | **Genes in Overlap** | **P-value** | **FDR** |
| --- | --- | --- | --- | --- | --- |
| **GO-CC** | | | | | |
| **Proteome** | | | | | |
| GO:1904724 | tertiary granule lumen | 55 | 3 | 6.41727E-07 | 0.000314446 |
| GO:0034774 | secretory granule lumen | 115 | 3 | 5.96944E-06 | 0.001462513 |
| GO:0031462 | Cul2-RING ubiquitin ligase complex | 15 | 2 | 9.39929E-06 | 0.001535217 |
| GO:0035580 | specific granule lumen | 62 | 2 | 0.000167823 | 0.020558293 |
| GO:0005856 | cytoskeleton | 407 | 3 | 0.000254786 | 0.024969057 |
| **GO-MF** | | | | | |
| **Proteome** | | | | | |
| GO:0050786 | RAGE receptor binding | 10 | 3 | 2.96108E-09 | 1.95727E-06 |
| GO:0048306 | calcium-dependent protein binding | 84 | 4 | 1.21659E-08 | 4.02083E-06 |
| GO:0001530 | lipopolysaccharide binding | 33 | 2 | 4.71091E-05 | 0.010379701 |
| GO:0005507 | copper ion binding | 55 | 2 | 0.00013196 | 0.021806456 |
| GO:0005546 | phosphatidylinositol-4 | 83 | 2 | 0.000300849 | 0.039772211 |
| **Transcriptome** | | | | | |
| GO:0004879 | nuclear receptor activity | 52 | 4 | 3.04E-05 | 0.020105379 |
| **GO-BP** | | | | | |
| **Proteome** | | | | | |
| GO:0019730 | antimicrobial humoral response | 56 | 4 | 2.32848E-09 | 4.03497E-06 |
| GO:0043312 | neutrophil degranulation | 484 | 6 | 5.60154E-09 | 4.03497E-06 |
| GO:0061844 | antimicrobial humoral immune response mediated by antimicrobial peptide | 69 | 4 | 5.46678E-09 | 4.03497E-06 |
| GO:0001816 | cytokine production | 23 | 3 | 4.35903E-08 | 2.35496E-05 |
| GO:0050832 | defense response to fungus | 28 | 3 | 8.05551E-08 | 3.48159E-05 |
| GO:0051092 | positive regulation of NF-kappaB transcription factor activity | 160 | 4 | 1.63358E-07 | 5.88363E-05 |
| GO:0042742 | defense response to bacterium | 199 | 4 | 3.90852E-07 | 0.000120662 |
| GO:0030593 | neutrophil chemotaxis | 78 | 3 | 1.85257E-06 | 0.000500426 |
| GO:0050729 | positive regulation of inflammatory response | 88 | 3 | 2.66706E-06 | 0.00064039 |
| GO:0002523 | leukocyte migration involved in inflammatory response | 13 | 2 | 6.98489E-06 | 0.001509435 |
| GO:0014002 | astrocyte development | 15 | 2 | 9.39929E-06 | 0.001846533 |
| GO:0051493 | regulation of cytoskeleton organization | 26 | 2 | 2.90344E-05 | 0.005211557 |
| GO:0002227 | innate immune response in mucosa | 27 | 2 | 3.13513E-05 | 0.005211557 |
| GO:0001817 | regulation of cytokine production | 38 | 2 | 6.26654E-05 | 0.008463739 |
| GO:2001244 | positive regulation of intrinsic apoptotic signaling pathway | 38 | 2 | 6.26654E-05 | 0.008463739 |
| GO:0031640 | killing of cells of other organism | 37 | 2 | 5.93781E-05 | 0.008463739 |
| GO:0002224 | toll-like receptor signaling pathway | 45 | 2 | 8.81352E-05 | 0.011203541 |
| GO:0019731 | antibacterial humoral response | 50 | 2 | 0.000108956 | 0.013080797 |
| GO:0006954 | inflammatory response | 389 | 3 | 0.000223158 | 0.025381286 |
| GO:0001503 | ossification | 76 | 2 | 0.000252284 | 0.027259232 |
| GO:0030307 | positive regulation of cell growth | 78 | 2 | 0.00026573 | 0.027344852 |
| GO:0050829 | defense response to Gram-negative bacterium | 83 | 2 | 0.000300849 | 0.029551557 |
| GO:0006919 | activation of cysteine-type endopeptidase activity involved in apoptotic process | 89 | 2 | 0.000345821 | 0.03249212 |
| **Transcriptome** | | | | | |
| hsa00564 | Glycerophospholipid metabolism | 97 | 5 | 2.12E-05 | 0.006850064 |
| **KEGG** | | | | | |
| **Proteome** | | | | | |
| hsa04657 | IL-17 signaling pathway | 93 | 3 | 3.15081E-06 | 0.001017711 |
| hsa05219 | Bladder cancer | 41 | 2 | 7.30545E-05 | 0.0117983 |
| hsa01522 | Endocrine resistance | 98 | 2 | 0.000419044 | 0.045117038 |
| **Transcriptome** | | | | | |
| hsa00564 | Glycerophospholipid metabolism | 97 | 5 | 2.12E-05 | 0.006850064 |
